# Vascular Endothelial Growth Factor Receptor-2 Couples Cyclo-Oxygenase-2 with Pro-Angiogenic Actions of Leptin on Human Endothelial Cells

**DOI:** 10.1371/journal.pone.0018823

**Published:** 2011-04-18

**Authors:** Elena Garonna, Kathleen M. Botham, Graeme M. Birdsey, Anna M. Randi, Ruben R. Gonzalez-Perez, Caroline P. D. Wheeler-Jones

**Affiliations:** 1 Department of Basic Sciences, Royal Veterinary College, University of London, London, United Kingdom; 2 Vascular Sciences Section, National Heart and Lung Institute, Imperial College London, Hammersmith Hospital, London, United Kingdom; 3 Morehouse School of Medicine, Atlanta, Georgia, United States of America; Ohio State University, United States of America

## Abstract

**Background:**

The adipocyte-derived hormone leptin influences the behaviour of a wide range of cell types and is now recognised as a pro-angiogenic and pro-inflammatory factor. In the vasculature, these effects are mediated in part through its direct leptin receptor (ObRb)-driven actions on endothelial cells (ECs) but the mechanisms responsible for these activities have not been established. In this study we sought to more fully define the molecular links between inflammatory and angiogenic responses of leptin-stimulated human ECs.

**Methodology/Principal Findings:**

Immunoblotting studies showed that leptin increased cyclo-oxygenase-2 (COX-2) expression (but not COX-1) in cultured human umbilical vein ECs (HUVEC) through pathways that depend upon activation of both p38 mitogen-activated protein kinase (p38^MAPK^) and Akt, and stimulated rapid phosphorylation of vascular endothelial growth factor receptor 2 (VEGFR2) on Tyr^1175^. Phosphorylation of VEGFR2, p38^MAPK^ and Akt, and COX-2 induction in cells challenged with leptin were blocked by a specific leptin peptide receptor antagonist. Pharmacological inhibitors of COX-2, the phosphatidylinositol 3-kinase (PI3K)/Akt pathway and p38^MAPK^ abrogated leptin-induced EC proliferation (assessed by quantifying 5-bromo-2′-deoxyuridine incorporation, calcein fluorescence and propidium iodide staining), slowed the increased migration rate of leptin-stimulated cells (in vitro wound healing assay) and inhibited leptin-induced capillary-like tube formation by HUVEC on Matrigel. Inhibition of VEGFR2 tyrosine kinase activity reduced leptin-stimulated p38^MAPK^ and Akt activation, COX-2 induction, and pro-angiogenic EC responses, and blockade of VEGFR2 or COX-2 activities abolished leptin-driven neo-angiogenesis in a chick chorioallantoic membrane vascularisation assay *in vivo*.

**Conclusions/Significance:**

We conclude that a functional endothelial p38^MAPK^/Akt/COX-2 signalling axis is required for leptin's pro-angiogenic actions and that this is regulated upstream by ObRb-dependent activation of VEGFR2. These studies identify a new function for VEGFR2 as a mediator of leptin-stimulated COX-2 expression and angiogenesis and have implications for understanding leptin's regulation of the vasculature in both non-obese and obese individuals.

## Introduction

Angiogenesis, the formation of new blood vessels from pre-existing vasculature, plays a central role in adult tissue homeostasis and is an important component of physiological and pathological processes including wound healing, tissue remodelling, tumour development and growth of atherosclerotic plaques [1]. Leptin is a cytokine derived principally from adipose tissue depots, including perivascular fat, and has multiple biological actions mediated through interaction with several alternatively spliced cell surface receptor isoforms. The long form (ObRb) is predominantly responsible for active signal transduction and functionally active ObRb has been identified in several peripheral cells and tissues, including human endothelial cells (EC) [2]. The functional significance of endothelial ObRb is highlighted by leptin's ability to regulate EC proliferation and apoptosis [3], [4], to stimulate nitric oxide synthesis [5], [6] and to promote angiogenesis [4], [7], [8], [9]. However, while leptin has been implicated as a regulator of both physiological and pathological angiogenesis and its pro-angiogenic properties are generally accepted [7], [10], the molecular basis of these actions is not yet defined and precisely how leptin regulates the pro-angiogenic functions of ECs remains unclear. Understanding these signalling mechanisms is of fundamental importance because aberrant signalling through ObRb in pathological conditions can lead to a state of hypothalamic leptin resistance which may extend peripherally to the endothelium.

A close association between inflammation and angiogenesis has recently been established and these processes are now recognised to play interdependent roles in orchestrating tissue repair and tumourigenesis. A role for leptin in inflammation is supported by reports of its direct modulation of monocyte, macrophage and T cell signalling and function [11], [12], [13]. In keeping with its ability to influence inflammatory processes and to promote angiogenesis, there is also evidence that leptin is chemotactic for both neutrophils [14] and endothelial cells [15]. One important pathway regulating the synthesis of key inflammatory molecules with potential importance in physiological/pathological angiogenic processes involves the cyclo-oxygenase (COX) enzymes. COX enzymes catalyse the committed step in prostanoid synthesis, converting free arachidonic acid into the prostaglandin (PG) precursors PGG_2_ and PGH_2_. The actions of tissue-specific terminal PG synthases [16] then catalyse the formation of prostanoids, including prostacyclin (PGI_2_), from PGH_2_. COX-1 is constitutively expressed by most cell types whereas COX-2 is generally absent, or present at low levels, but can be induced by pro-inflammatory and mitogenic stimuli [17], [18]. Leptin, for example, has been documented to promote COX-2 induction in both macrophage and adenocarcinoma cell lines [11], [19]. There is also growing evidence for the involvement of COX-2-derived mediators in angiogenic processes, particularly tumour angiogenesis, and in keeping with this role non-steroidal anti-inflammatory drugs that selectively block COX-2 activity have both anti-angiogenic and anti-carcinogenic actions [20], [21]. The potential effects of leptin on pro-inflammatory signalling and gene expression in human ECs have received little attention, but regulation of COX-2 induction and activity could represent a key mechanism contributing to leptin's angiogenic actions.

Here, we have investigated the relationship between pro-inflammatory and pro-angiogenic actions of leptin in human ECs. We show that leptin enhances endothelial COX-2 expression through mechanisms that depend upon activation of p38^MAPK^ and phosphatidylinositol 3-kinase (PI3K)/Akt. We also provide evidence that the activities of p38^MAPK^, PI3K/Akt and COX-2 are required for leptin-mediated angiogenic activity and that phosphorylation of vascular endothelial growth factor receptor 2 (VEGFR2) on Tyr^1175^ is upstream of leptin-induced p38^MAPK^/PI3K/Akt activity, COX-2 induction and the associated angiogenic responses. Together, these studies suggest that a functional endothelial p38^MAPK^/Akt/COX-2 signalling axis is required for leptin's pro-angiogenic actions and that this is mediated, at least in part, by ObRb-dependent activation of VEGFR2. These studies highlight the importance of leptin as a direct modulator of EC function and suggest a novel interaction between leptin- and VEGF-induced signalling in human ECs that has functional significance for the regulation of leptin's angiogenic activity.

## Materials and Methods

### Ethics Statement

Human umbilical cord collection (obtained with informed written consent) conformed to the principles outlined in the Declaration of Helsinki and was approved by East London and The City Local Research Ethics Committee (ref. no. 04/Q0604/4). Experiments conducted on the developing extra-embryonic vasculature (chorioallantoic membrane; CAM) of the chicken embryo commenced and were completed before day 10 of development and thus did not require ethical approval.

### Materials

Human recombinant leptin was purchased from Peprotech (Rocky Hill, USA). Human α-thrombin, bovine serum albumin (BSA; fraction v), and polyvidinylidene membranes (Immobilon-p^TM^) were all purchased from Sigma (Dorset, UK). Human recombinant VEGF-A_165_ and the VEGFR2 blocking antibody were from R&D Systems (Oxford, UK). Endothelial cell growth factor (ECGF) was from Upstate (Millipore; Durham, UK) and growth factor-reduced Matrigel was obtained from BD Biosciences (Oxford, UK). SB202190, NS398, LY294002 and SU4516 were all from Calbiochem (Nottingham, UK). Polyclonal COX-1, COX-2, phospho-Akt (Ser^473^) and p38^MAPK^ antibodies were all purchased from Santa Cruz Biotechnology (Santa Cruz, CA) and the ObRb (rabbit polyclonal) antibody was from Abcam (Cambridge, UK). Anti-phospho-p38^MAPK^ antibody was purchased from Biosource (Nivelles, Belgium), and antibodies against phosphorylated VEGFR2 (Tyr^1175^), VEGFR2, phospho-tyrosine, phospho-GSK3β and Akt were from Cell Signalling Technology (Hertfordshire, UK). Horseradish peroxidise-conjugated goat anti-rabbit and rabbit anti-goat antibodies were obtained from Pierce (Rockford, USA). Reagents for SDS-polyacrylamide gel electrophoresis (SDS-PAGE) were purchased from Bio-Rad (Hemel Hempstead, Hertfordshire, UK) and National Diagnostics (Hessle, Hull, UK). The bicinchonic acid (BCA) protein assay was from Pierce (Rockford, USA). Culture media were purchased from Sigma, PAA Laboratories (Somerset, UK) or BDH. The BrdU ELISA kit was purchased from Roche Diagnostics (Mannheim, Germany), calcein AM and propidium iodide from Invitrogen (Paisley, UK), and all other reagents from Sigma or BDH at the equivalent of AnalaR grade. The leptin peptide receptor antagonist 2 (LPrA2) was synthesised and purified as described elsewhere [22] and dissolved in a sterile filtered vehicle solution (0.0025% dimethyl sulfoxide in PBS).

### Endothelial cell (EC) culture

Human umbilical vein ECs (HUVEC) were isolated and cultured as described in previous publications [18], [23]. Briefly, cells were grown in medium M199 supplemented with 20% FCS, 4 mM glutamine, 100 units/mL penicillin, 100 units/mL streptomycin and 20 mM NaHCO_3_ and cultured at 37°C in 5% CO_2_/95% air in 25 mm^2^ tissue culture flasks pre-coated with 1% gelatin (w/v). When confluent, cells were passaged into 75 mm^2^ tissue culture flasks and cultured in M199 (as above) containing 20 µg/mL ECGF. Unless stated otherwise, cells were used routinely at passage 2. Experimental incubations were carried out in Hepes-buffered medium M199 with or without 10% FCS and 10 µg/mL ECGF as indicated.

### Stimulation of ECs with leptin

In all experiments ECs were exposed to human recombinant leptin at final concentrations of 1, 10 and 100 ng/mL for the times indicated in the figure legends, and the effects of leptin compared directly to those of VEGF_165_ (25 ng/mL). The leptin concentration evoking a maximal effect on the signalling and functional responses investigated differed among HUVEC isolates (data not shown), demonstrating that primary human ECs display variable sensitivity to leptin. Despite this variation, the level of stimulation observed was highly consistent across experiments. Thus, values used to generate the means for statistical analyses were those representing the maximum response to leptin for each experiment within a group (n = 3–15 individual cell isolates).

### Quantification of cell proliferation using propidium iodide staining

HUVEC (20,000/well) were seeded onto 24-well plates and cultured for 6–14 hours. Cells were starved (12–16 hours) in serum- and ECGF-free M199 and subsequently exposed to agonists prepared in Hepes-buffered M199 as detailed in the figure legends. Following incubation at 37°C for 24 hours, the medium was removed, the cells washed twice with PBS/0.5% BSA and then fixed in PBS/4% paraformaldehyde. Fixed cells were washed in PBS and a solution of propidium iodide (1 ng/mL in PBS) was added (500 µL/well). Nuclei were visualised using a Zeiss LSM 510 confocal microscope and 12 fields per well were analysed using a purpose-designed macro.

### Measurement of VEGF_165_ release

VEGF_165_ in supernatants from HUVEC exposed to leptin (1–100 ng/mL) for times ranging between 5 minutes and 24 hours was measured by sandwich ELISA. 96-well microtiter plates were coated with 100 µL/well of goat anti-human VEGF_165_ (0.4 µg/mL) buffered with 50 mM sodium bicarbonate (pH 9.6) and incubated overnight at 4°C. Plates were then incubated with 1% BSA in PBS for 1 hour at room temperature to restrict non-specific binding. Human recombinant VEGF_165_ standards or samples were added to the wells (100 µL/well) and incubated for 2 hours at room temperature. Biotinylated goat anti-human VEGF_165_ (0.2 µg/mL; 50 µL/well) was added and incubations carried out for 2 hours at room temperature. A colour reaction was induced by the addition of tetramethylbenzidine/hydrogen peroxide substrate solution (100 µL/well) and was stopped 30 minutes later by addition of 1 M sulphuric acid (50 µL/well). The optical density (OD) at a wavelength of 405 nm was then measured using a Wallac Victor^2^ 1420 multilabel counter. The OD of the standards in serum-free medium (0.25–5 ng/mL) was used to calculate the concentration of VEGF_165_ in the sample.

### Western blotting

Confluent HUVEC in 60 mm^2^ dishes were serum- and ECGF- starved for 16 hours. Quiescent cells were then subjected to treatments as detailed in the figure legends. Whole cell lysates were prepared and analysed by SDS-PAGE and immunoblotting as described [23]. Immunoreactive proteins were visualised by enhanced chemiluminescence and densitometric analysis of immunoblots was performed using a Bio-Rad scanning densitometer and Quantity One analyzing software.

### Immunoprecipitation

Confluent HUVEC in 60 mm^2^ dishes were serum-starved (10–12 hours), treated with vehicle, leptin or VEGF (5 minutes) and lysed on ice in a buffer (pH 7.4) composed of 50 mM Hepes, 10 mM sodium pyrophosphate, 100 mM NaF, 2 mM EDTA, 2 mM Na_3_VO_4_, 1% Triton (v/v), 10% glycerol (v/v), 0.5 mM phenylmethanesulphonylfluoride, 10 µg/mL aprotinin and 10 µg/mL leupeptin. Equal quantities of cell lysates (200 µg) were incubated (4°C) with VEGFR2 antibody (2 µg) overnight, with rotation. Protein A-sepharose (50 µL) diluted 1∶1 in PBS was then added and rotation continued for 1 hour. The resulting immunoprecipitates were washed three times by centrifugation followed by resuspension in ice-cold PBS. Sample buffer (20 µL; Tris HCL 100 mM (pH 6.8), 20% (v/v) glycerol, 4% (w/v) SDS, 0.1% (w/v) bromophenol blue, 10% (v/v) β-mercaptoethanol) was added to the precipitates prior to analysis by SDS-PAGE and western blotting.

### Wound-migration assay

An *in vitro* wounding assay was used to measure directional EC migration. HUVEC were seeded onto gelatin-coated 24-well plates and allowed to form confluent monolayers. Cells were then cultured in reduced serum/growth factor medium (M199: 10% FCS; 10 mg/mL ECGF) overnight and pre-treated with the appropriate agonist in 10% FCS and 5 mg/mL ECGF in M199 for 6 hours. Monolayers were scratch wounded using a sterile 200 µl pipette tip. Cells were washed with PBS and agonists/inhibitors added in 0.5 mL of medium containing 10% FCS and 5 mg/mL ECGF. The plate was then placed in the temperature-controlled chamber (37°C; 95% air, 5% CO_2_) of a Zeiss confocal microscope (LSM 510 Meta Axiovert 200 M). The software was programmed to capture an image of each well at the same wound location every 30 minutes for 15–19 hours and the rate of migration assessed by measurements of wound width using the Zeiss software.

### Matrigel assay

Measurements of capillary-like tube formation by HUVEC were achieved using an *in vitro* assay of EC differentiation on Matrigel. HUVEC were cultured in M199 containing 5% FCS and 5 mg/mL ECGF overnight. Growth factor-reduced Matrigel was plated onto 96-well plates (65 µL/well) and incubated at 37°C for 30 minutes. HUVEC were seeded at 10,000/well (in M199 containing 5% FCS/5 mg/mL ECGF; 200 µL/well and the appropriate experimental treatments), in duplicate. After 8 hours the medium was removed, the cells washed twice in PBS and fixation carried out in 4% paraformaldehyde (in PBS). Images were captured using a Leica DMIRB inverted microscope. Tube formation was quantified by counting the total number of interbranches in each well using Leica QWin Imaging software.

### Chick chorioallantoic membrane (CAM) assay

To assess the roles of VEGFR2 and COX-2 activities in leptin-mediated angiogenesis *in vivo* we used our chick CAM vascularisation assay [24]. Fertilised white Leghorn eggs were incubated at 37°C in a humidified incubator and windowed. On day 7 of development, sterile filters soaked with either vehicle, leptin (1 µg/disk) or VEGF (100 ng/disk) in the presence or absence of SU5416 or NS398 (10 µg/disk) were applied to relatively avascular regions of the CAM. CAMs were fixed (4% paraformaldehyde in PBS) *in ovo* on day 9 and photographed in the localised area of the filter. The newly capillarised area in the region of each filter was quantified using Leica QWin Lite software and neovascularisation is expressed as an angiogenic index (n = 12–15 eggs per treatment).

### Statistical analysis

One-way ANOVA, repeated measures ANOVA (with Bonferroni *post hoc* test) or unpaired Student's t test, as appropriate, were used to compare means of groups of data using GraphPad Prism version 5. Within each experiment treatments were performed in triplicate and the average value treated as a single data point. Data are expressed as mean ± SEM, where *n* is the number of individual experiments, each performed on a different HUVEC isolate; *p* values equal to or less than 0.05 on two-sided tests were considered statistically significant.

## Results

### Leptin promotes COX-2 expression and prostanoid synthesis in human ECs

**Figure 1:**
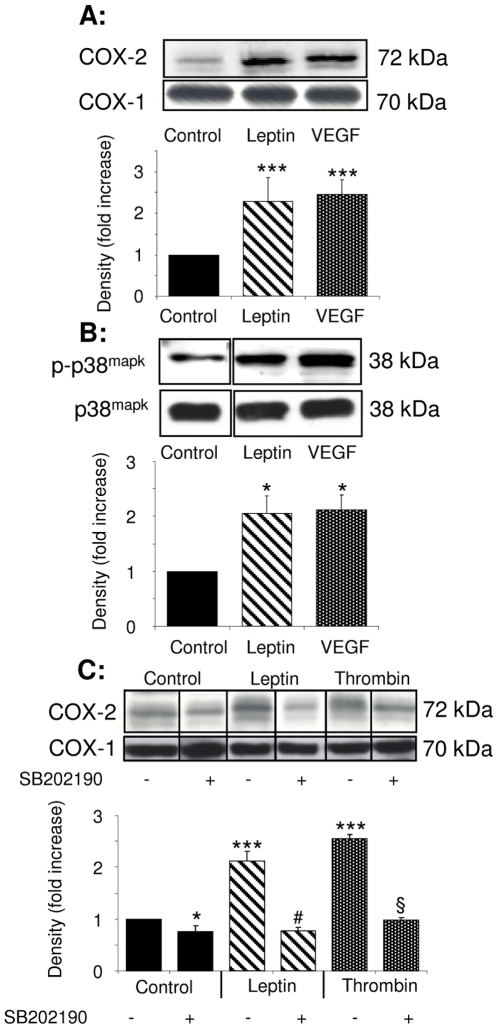
Leptin increases COX-2 expression in HUVEC in a p38^MAPK^-dependent manner. Cells were exposed to vehicle alone, leptin (100 ng/mL) or VEGF (25 ng/mL) for 6 hours (A) or 10 mins (B). Lysates were analysed by SDS-PAGE and immunoblotting with COX-2 or COX-1 antibodies (A) and for p38^MAPK^ activation using an anti-phospho(p)-p38^MAPK^ antibody (B). Immunoblots in A are each representative of 15 separate experiments on cells from 15 umbilical veins. Data from densitometric analyses of COX-2 expression are given as mean ± SEM (n = 15). *** *p*<0.001 *versus* control. B. Densitometric analyses of p-p38^MAPK^ expression from 5 experiments (mean ± SEM. * *p*<0.05 *versus* controls). C. HUVEC were pre-treated for 30 minutes with SB202190 (1 µmol/L) and then exposed to vehicle, leptin or thrombin (1 U/mL) in the continued absence or presence of SB202190 for 6 hours. Analysis of immunoblots from 3 experiments is shown (mean ± SEM: * *p*<0.05; ***p<0.001 *versus* controls; # *p*<0.05 *versus* leptin; § *p*<0.05 *versus* thrombin).

To determine whether leptin triggers pro-inflammatory pathways in ECs we initially examined COX enzyme expression. Leptin (1–100 ng/mL) had no effect on COX-1 expression (Figure 1A) but elicited a time-dependent induction of COX-2 mRNA (Figure S1 and Text S1) and protein with maximal up-regulation of protein levels evident after 6 hours and comparable to that induced by VEGF-A_165_ (VEGF; 25 ng/mL) (Figure 1A) [17]. These changes were accompanied by enhanced synthesis of 6-keto-PGF_1α_ and PGE_2_, but not TxB_2_ (Figure S2), demonstrating that leptin increases endothelial COX-2 expression and stimulates prostanoid synthesis and release.

### Leptin-induced COX-2 expression is regulated by p38^MAPK^ and Akt

COX-2 expression in ECs depends upon MAPK signalling pathways with p38^MAPK^ playing a prominent role [25]. Leptin enhanced p38^MAPK^ phosphorylation (Figure 1B) in a time-dependent manner (data not shown) with maximal activation evident after 10 minutes. The selective p38^MAPK^ inhibitor SB202190 concentration-dependently decreased COX-2 protein expression in leptin- and thrombin-stimulated cells (Figure 1C; concentration data not shown). In addition, in keeping with our recent findings [26], [27], HUVEC exhibited some basal COX-2 expression which was also reduced by SB202190 treatment (data not shown). These data show that leptin-induced COX-2 expression, in common with induction by other pro-inflammatory agonists, is regulated by p38^MAPK^ activation.

**Figure 2:**
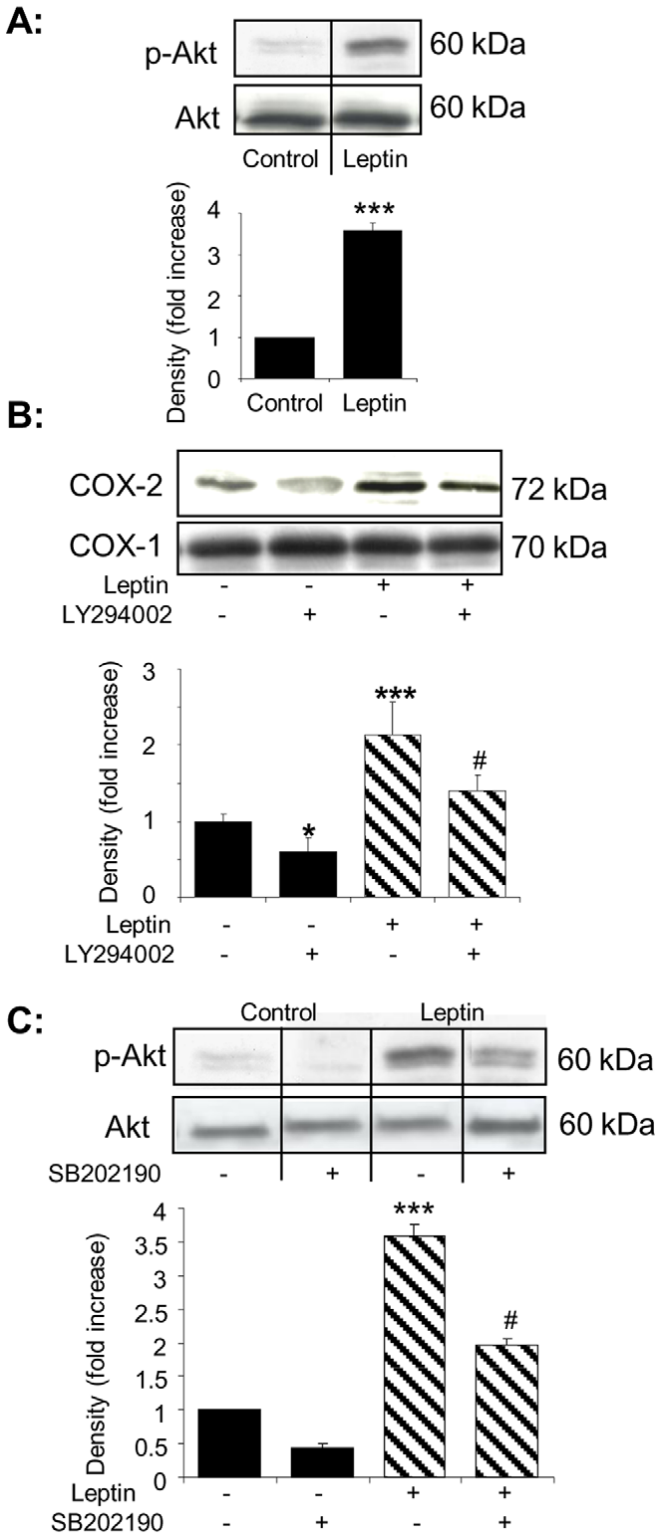
Akt regulates leptin-induced COX-2 expression. A. HUVEC were exposed to either vehicle alone or leptin (50 ng/mL) for 30 minutes and cell lysates monitored for Akt activation using a phospho-Akt antibody. Histograms show densitometric analysis of immunoblots from 5 individual experiments (mean ± SEM). B. Cells were exposed to vehicle or LY294002 (1 µmol/L) for 30 minutes and then challenged with leptin in the continued absence or presence of LY294002 for 6 hours. Analyses of immunoblots from 3 separate experiments are shown (mean ± SEM). * *p*<0.05, *** *p*<0.001 *versus* controls; # *p*<0.05 *versus* leptin. C. HUVEC were pre-treated with either vehicle or SB202190 (1 µmol/L) for 30 minutes and then incubated with vehicle (C) or leptin in the presence or absence of SB202190 for a further 30 minutes. Histograms show combined analysis of immunoblots probed with a phospho-Akt antibody (n = 3 individual experiments; mean ± SEM). *** *p*<0.001 *versus* controls; # *p*<0.05, ### *p*<0.001 *versus* leptin.

There is evidence that PI3K/Akt may be important in mediating some of leptin's peripheral effects [6], [15], [28] so we determined whether leptin activates Akt in ECs and investigated its potential role in COX-2 induction. Akt phosphorylation was enhanced in cells exposed to leptin (Figure 2A) and LY294002, a PI3K inhibitor which blocks downstream Akt phosphorylation (data not shown), reduced leptin-induced COX-2 expression and inhibited basal COX-2 expression (Figure 2B). p38^MAPK^ blockade with SB202190 also decreased leptin-induced Akt phosphorylation (Figure 2C). Thus, PI3K/Akt and p38^MAPK^ activities regulate COX-2 induction in leptin-stimulated ECs.

### Pro-angiogenic functions of leptin-stimulated ECs are p38^MAPK^-, Akt- and COX-2-dependent

**Figure 3:**
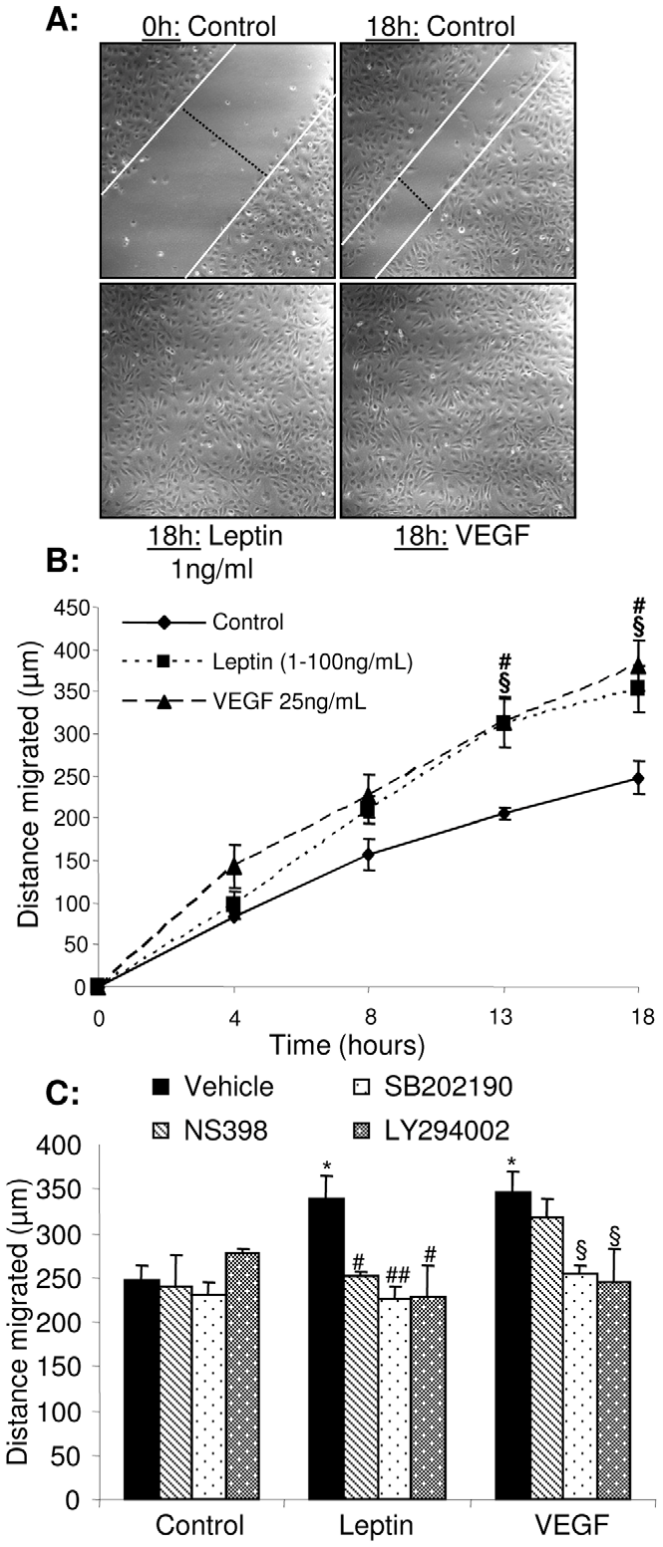
Leptin-stimulated endothelial cell migration is attenuated by blockade of p38^MAPK^, Akt or COX-2 activities. A. Confluent monolayers of quiescent HUVEC were scratch wounded and treated with either vehicle (control), leptin (1–100 ng/mL) or VEGF (25 ng/mL). The rate of wound closure was monitored in real time by confocal microscopy (B). C. Confluent cells were exposed to vehicle, leptin or VEGF in the presence or absence of NS398, SB202190 or LY294002 (1 µmol/L), wounded, and migration monitored (18 h). Data in B and C are mean ± SEM of 3 separate experiments with duplicate observations per treatment. * *p*<0.05 *versus* controls; # *p*<0.05, ## *p*<0.005 *versus* leptin; § *p*<0.05 cells *versus* VEGF.

**Figure 4:**
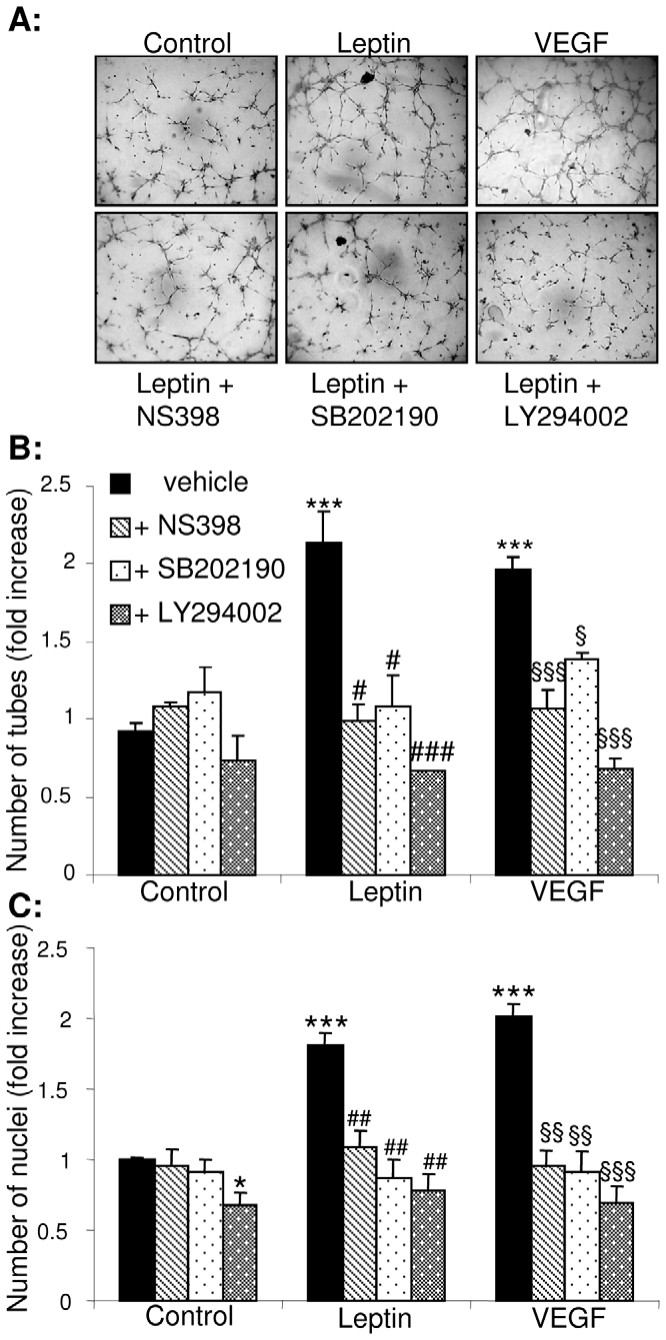
Leptin-induced capillary-like tube formation on matrigel requires activation of p38^MAPK^ and Akt, and is COX-dependent. HUVEC were cultured on growth-factor reduced matrigel and treated with either vehicle, leptin (1–100 ng/mL) or VEGF (25 ng/mL) in the presence or absence of 1 µmol/L NS398, SB202190 or LY294002 for 8 hours. Cells were photographed (magnification: ×5) and the number of tubes counted. Each treatment was carried out in duplicate in 3 separate experiments. A. Representative images; B. Analysis of tube number expressed as fold increase (± SEM) compared to control. *** *p*<0.001 *versus* control; # *p*<0.05, ### *p*<0.001 *versus* leptin; § *p*<0.05, §§§ *p*<0.001 *versus* VEGF. C. HUVEC were exposed to vehicle, leptin or VEGF in the presence or absence of NS398, SB202190 or LY294002 for 24 hours. Data are expressed as fold increase in number of nuclei (n = 4 experiments) with duplicate observations per treatment. *p<0.05, *** *p*<0.001 *versus* controls; ## *p*<0.005 *versus* leptin; §§ *p*<0.005, §§§ *p*<0.001 *versus* VEGF.

We next investigated the importance of these pathways for EC migration, differentiation and proliferation. In an *in vitro* wound assay leptin (1–100 ng/mL) enhanced the rate of wound closure to a similar extent to that observed in VEGF-treated cultures (Figure 3A and B). Studies with selective pharmacological inhibitors of COX-2 (NS398), p38^MAPK^ (SB202190) and PI3K/Akt (LY294002) showed that EC migration under basal conditions was unaffected by inhibitor treatment, whereas leptin-induced migration was reduced by all three treatments (Figure 3C). SB202190 and LY294002, but not NS398, also significantly decreased VEGF-induced migration (Figure 3C). Capillary-like tube formation on Matrigel by leptin-stimulated HUVEC was equivalent to that observed in cells exposed to VEGF, and both leptin- and VEGF-induced responses were reduced in the presence of NS398, SB203580 and LY294002 (Figure 4A and 4B). Similarly, measurements of BrdU incorporation, mitochondrial enzyme activity status and nuclear propidium iodide staining showed that leptin and VEGF enhanced EC proliferation to similar extents (Figure S3) and that leptin- and VEGF-driven proliferation were abrogated by exposure to NS398, SB202190 or LY294002 (Figure 4C). Together, these data show that activation of p38^MAPK^ and Akt, as well as COX-2 activity, regulate leptin-induced EC migration, proliferation and differentiation.

### Leptin treatment stimulates VEGFR2 phosphorylation but not VEGF release

**Figure 5:**
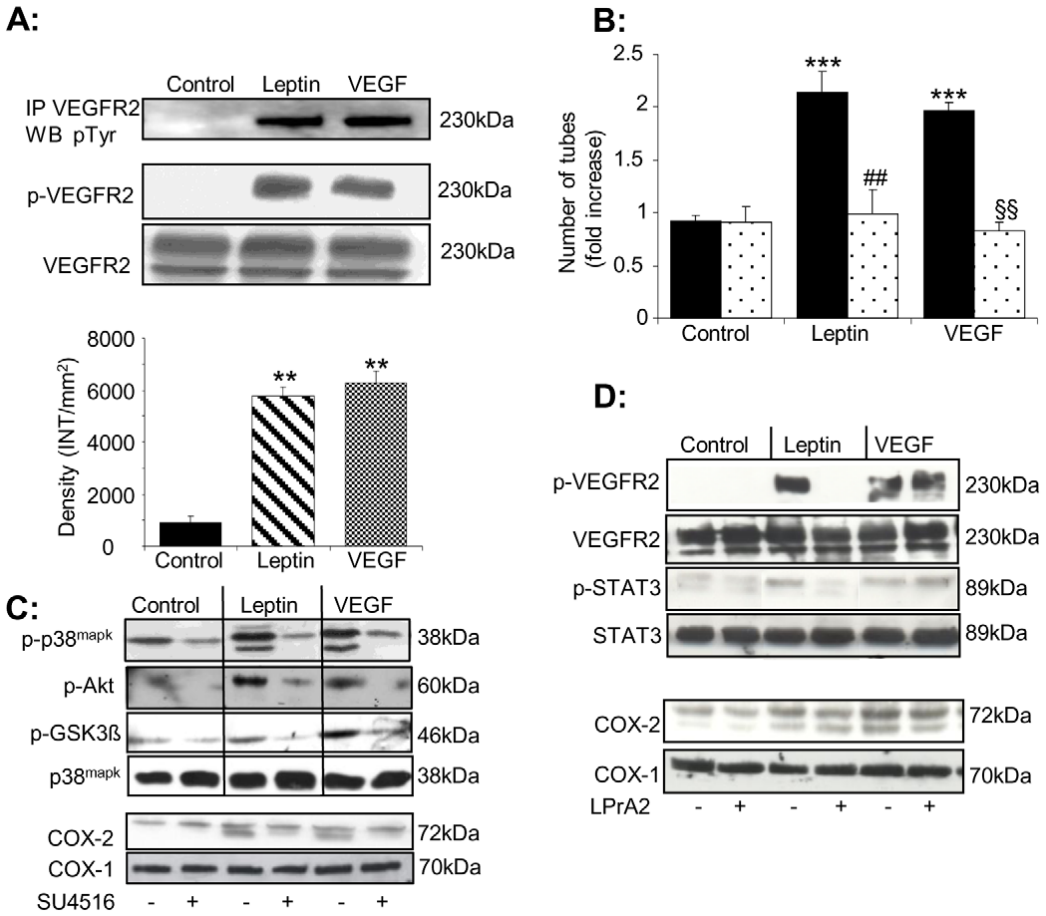
VEGFR2 mediates leptin-stimulated activation of p38^mapk^ and Akt, COX-2 induction and downstream angiogenic responses. A. VEGFR2 was immunoprecipitated from vehicle-, leptin (1 ng/mL)- or VEGF (25 ng/mL)-treated HUVEC (5 minutes) and immunoblots probed with an anti-phospho(p)-tyrosine antibody. The blot shown is representative of 3 individual experiments. Whole cell lysates were also analysed by immunoblotting with phospho(p)-VEGFR2 or total VEGFR2 antibodies. Densitometric analyses of p-VEGFR2 immunoblots from 3 separate experiments are shown (** *p*<0.01 *versus* control). B. Tube formation on matrigel following treatment (8 hours) with vehicle, leptin (1–100 ng/mL) or VEGF (25 ng/mL) in the presence or absence of SU4516 (5 µmol/L). * *p*<0.05 and *** *p*<0.001 *versus* control; # *p*<0.05 and ## *p*<0.005 *versus* leptin; § *p*<0.05 and §§§ *p*<0.005 *versus* VEGF. C. HUVEC were pre-treated with vehicle or SU4516 (5 µmol/L) and then incubated with leptin (10 ng/mL) or VEGF (25 ng/L) in the continued presence or absence of SU4516 for 10 mins or 6 hours. Blots were probed with antibodies against phospho(p)-p38^mapk^, phospho-Akt, phospho-GSK3β or COX-2 and are representative of 3 separate experiments. D. HUVEC were exposed to vehicle, leptin (1 ng/mL) or VEGF (25 ng/mL) in the presence or absence of the ObR antagonist LPrA2 (30 µmol/L) for 5 mins or 6 hours. Lysates were analysed by SDS-PAGE and immunoblotting for phosphorylated forms of VEGFR2 and Stat-3, total VEGFR2 and Stat-3, or COX-1 and -2. Data are representative of 2–3 individual experiments.

The equivalency of leptin and VEGF's effects prompted us to investigate whether leptin utilises VEGFR2 to affect signalling and functional responses. A 5 minute exposure to leptin (1–100 ng/ml) increased VEGFR2 phosphorylation (Tyr^1175^) to a level similar to that observed in VEGF-treated cells (Figure 5A). To determine whether leptin-stimulated VEGFR2 Tyr^1175^ phosphorylation depends upon VEGF release from ECs we measured the VEGF concentrations in medium from leptin-stimulated cells by ELISA. No significant VEGF was detected in medium from cells exposed to leptin for 5 minutes, 1 hour, 6 hours or 24 hours (not detectable, not detectable, 128±109 and 105±99 pg/mL/mg protein, respectively; n = 3 individual experiments). Thus, leptin promotes rapid phosphorylation of VEGFR2 on Tyr^1175^ in the absence of detectable VEGF release.

### Role of VEGFR2 phosphorylation in leptin-driven responses

To examine the role of leptin-stimulated VEGFR2 phosphorylation in ECs we used SU5461, an established inhibitor of VEGFR2 tyrosine kinase activity [29]. SU4516 (5 µmol/L) inhibited leptin- and VEGF-induced proliferation but did not modify basal (Figure S4) or hepatocyte growth factor (HGF)-induced proliferation (data not shown). Additionally, SU4516 (Figure 5B) and a VEGFR2 blocking antibody (Figure S5) reduced leptin- and VEGF-stimulated tube formation, and SU4516 abrogated the increased migration rate evident in leptin- and VEGF-stimulated ECs (Figure S4). Leptin- and VEGF-induced phosphorylation of p38^MAPK^ and Akt, and of GSK3β (an established Akt substrate), as well as induction of COX-2 were also attenuated by SU4516 (Figure 5C). These data strongly suggest that leptin stimulates VEGFR2 activity to promote intracellular signalling and COX-2 induction, leading to increased EC proliferation, motility and differentiation.

### An ObRb antagonist attenuates leptin's effects on VEGFR2 phosphorylation and downstream signalling

To determine whether binding of leptin to ObRb is required for leptin-stimulated VEGFR2 Tyr^1175^ phosphorylation we used a leptin peptide receptor antagonist (LPrA2) [22]. We confirmed that HUVEC express ObRb (Figure S6) and showed that in the presence of LPrA2 (30 µmol/L), leptin failed to promote VEGFR2 phosphorylation while VEGF-induced receptor phosphorylation was unaffected (Figure 5D). It is well established that leptin binding to ObRb activates JAK-STAT signalling so to confirm blockade of leptin binding to ObRb we monitored Stat-3 phosphorylation. In cells treated with LPrA2 leptin did not promote Stat-3 phosphorylation while VEGF-induced Stat-3 phosphorylation was still evident (Figure 5D). Similarly, exposure to LPrA2 reduced leptin-stimulated COX-2 induction without affecting the response to VEGF. These data indicate that leptin-mediated VEGFR2 phosphorylation and its downstream effects require binding to ObRb. Leptin-induced VEGFR2 phosphorylation does not result from direct receptor association since ObRb was absent from VEGFR2 immunoprecipitates prepared from control, leptin- and VEGF-stimulated ECs (Figure S6).

### Blockade of VEGFR2 and COX-2 activities inhibits leptin-stimulated angiogenesis *in vivo*

**Figure 6:**
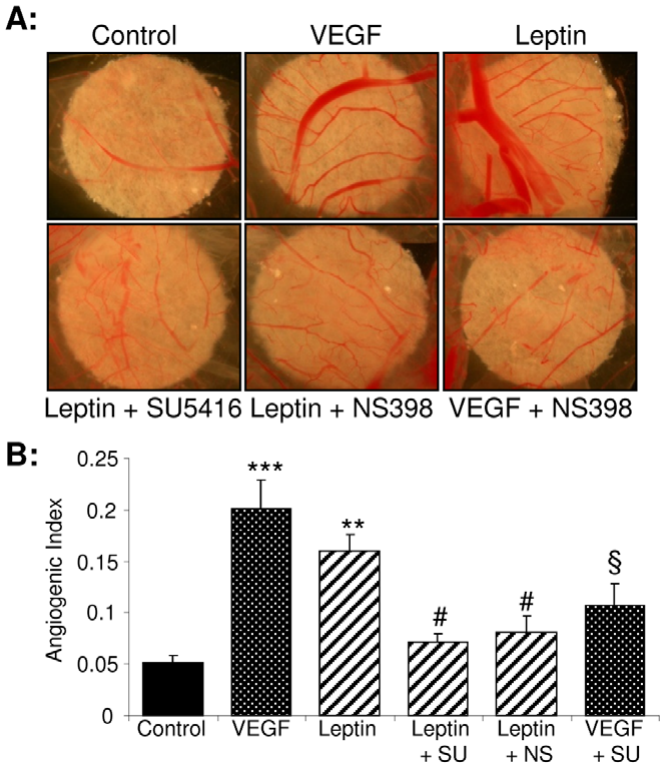
Leptin-induced angiogenesis *in vivo* is attenuated by blockade of COX-2 and VEGFR2 activities. Fertilised eggs were incubated and windowed. On day 7 of development sterile filters soaked with vehicle, leptin or VEGF in the absence or presence of SU5416 (SU) or NS398 (NS) were applied to the CAMs (see Methods) which were photographed 48 hours later; representative images are shown. Pooled data from 12–15 eggs per treatment are given as angiogenic index (mean ± SEM).

To investigate the biological significance of leptin-stimulated VEGFR2 phosphorylation and COX-2 induction for leptin's angiogenic actions *in vivo* we monitored neo-angiogenesis using our chick CAM vascularisation assay [24] (Figure 6). Leptin strongly stimulated new capillary formation, to an extent comparable to that of VEGF, and this was attenuated by blockade of VEGFR2 activity with SU4516 or by selective inhibition of COX-2 activity with NS398. These results confirm the relevance of leptin-stimulated VEGFR2 phosphorylation and COX-2 activity for leptin-driven angiogenesis *in vivo*.

## Discussion

Leptin is a pleiotropic adipokine with pro-angiogenic actions mediated by poorly characterised cellular mechanisms. In this study we tested the overarching hypothesis that the angiogenic behaviours of leptin-stimulated human ECs depend upon COX-2 activity and require VEGFR2 activation. To test this hypothesis we investigated whether leptin modifies COX isoform expression and through which signalling pathways, determined whether this signalling axis is utilised by leptin to promote angiogenic actions in ECs, and explored the potential functional interaction between leptin-mediated signalling and VEGFR2 activation *in vitro* and *in vivo*. We demonstrated that: (i) leptin induces endothelial COX-2, but not COX-1 expression; (ii) leptin activates p38^MAPK^ and PI3K/Akt pathways and both are utilised to regulate COX-2 expression; (iii) leptin enhances proliferation, directional migration and differentiation of ECs through activation of p38^MAPK^/Akt signalling and through increased COX-2 activity; (iv) leptin causes rapid VEGFR-2 phosphorylation upstream of p38^MAPK^/Akt/COX-2 and this is required for leptin's functional effects on ECs and for leptin-stimulated neo-angiogenesis *in vivo*.

In accordance with our previous studies [17], [18] we confirmed that VEGF induces endothelial COX-2 expression and show, for the first time to our knowledge, that leptin at physiological concentrations promotes COX-2 induction and prostanoid synthesis, notably PGI_2_ and PGE_2_, by human ECs and is equivalent to VEGF in its capacity to enhance these responses. Cellular regulation of COX-2 expression occurs in a cell- and context-specific manner and several signalling pathways have been implicated. We and others have shown that MAPKs, including p38^MAPK^, regulate COX-2 expression in ECs exposed to physiological and pathological stimuli [21], [25], [27] and leptin has been reported to influence p38^MAPK^ activity in non-vascular cell types [30], [31]. Our data show that leptin caused robust p38^MAPK^ activation in ECs, and since selective pharmacological blockade of p38^MAPK^ activity abrogated COX-2 induction in leptin-stimulated cells, COX-2 expression in this setting is p38^MAPK^-dependent. There is also evidence that leptin triggers activation of the PI3K/Akt pathway in ECs [15], but whether this is utilised to regulate COX-2 induction and/or angiogenic responses has not been addressed. We show that leptin enhanced Akt phosphorylation and that inhibition of PI3K activity reduced COX-2 induction, suggesting that p38^MAPK^ and PI3K/Akt signalling underlies the increased COX-2 expression in leptin-stimulated ECs. We have previously shown that cross-talk between MAPK pathways and other signalling events is important for regulating andothelial COX-2 expression and prostanoid synthesis, as well as for functional responses in other cell types [27], [32]. In this study, inhibiting p38^MAPK^ activity with SB202190 reduced Akt phosphorylation in leptin- and thrombin-stimulated ECs, implying that p38^MAPK^ activation is upstream of Akt in agonist-challenged ECs and suggesting that interaction between p38^MAPK^ and PI3K/Akt signalling [33] has potential importance for control of endothelial COX-2 induction.

Recent studies have described context-specific roles for both p38^MAPK^ and Akt in cell growth [19], [21], [34] but the functional significance of these pathways in leptin-stimulated ECs is unknown. Here, we examined the importance of p38^MAPK^, PI3K/Akt and downstream COX-2 activity for *in vitro* angiogenic responses of leptin-stimulated ECs. We showed that key components of angiogenesis (EC proliferation, migration and differentiation) were abrogated by inhibition of p38^MAPK^ and PI3K/Akt, providing clear evidence for the involvement of these signalling pathways in leptin-stimulated EC growth and motility. *In vitro* angiogenic responses were shown to be COX-2-dependent since selective inhibition of COX-2 activity with NS398 reduced the extent of EC proliferation, migration and differentiation in the presence of leptin. Our results therefore provide strong evidence that leptin's angiogenic actions in primary ECs depend upon an intact p38^MAPK^/Akt/COX-2 signalling axis. Although very few studies have addressed the importance of COX enzymes for leptin's cellular actions, one previous study reported that NS398 had no effect on EC differentiation on Matrigel [35], a finding which most likely reflects the use of very high passage ECs with an altered phenotype. In our study, COX-2 blockade also suppressed leptin-stimulated neo-angiogenesis in an *in vivo* vascularisation assay, further emphasizing the physiological relevance of COX-2 activity for leptin's angiogenic activity.

Binding of growth factors to their cognate receptors elicits a range of biological functions in ECs. Pro-angiogenic signalling is mediated principally through VEGFR2, the primary VEGFR expressed by vascular ECs. Previous reports indicated that leptin increases VEGF synthesis and VEGFR2 expression by breast cancer cells [31] and suggested that leptin and VEGF can cooperate to promote angiogenesis *in vivo* [8], but the molecular basis of this interaction is not characterised. Here, we noted that leptin's effects on COX-2 induction and the angiogenic properties of ECs were both qualitatively and quantitatively equivalent to those of VEGF, leading us to hypothesise that leptin regulates EC function through VEGFR2 activation. We found that leptin treatment caused rapid phosphorylation of VEGFR2 (Tyr^1175^) with maximal phosphorylation evident after 5 minutes. Since leptin did not promote VEGF release from ECs, and ObRb and VEGFR2 did not co-immunoprecipitate, our data collectively suggest that leptin-mediated VEGFR2 phosphorylation most likely occurs independently of exogenous VEGF and in the absence of direct ObRb:VEGFR2 association. These findings support the recent suggestion that EC-derived VEGF does not contribute to the angiogenic response through autocrine actions [36], [37] and indicate that leptin may regulate receptor phosphorylation intracellularly and/or through mechanisms that are independent of extracellular VEGF-A_165_ binding to VEGFR2.

There is evidence for both ligand-dependent and -independent transactivation of VEGFR2 by inflammatory and angiogenic agonists [37], [38], [39]. Here, the requirement for leptin-ObRb interaction to enable VEGFR2 activation is demonstrated by our observation that treatment with LPrA2, a specific ObRb blocking peptide [22], abolished leptin-stimulated VEGFR2 and Stat-3 phosphorylation and reduced COX-2 induction, but did not modify Stat-3 or VEGFR2 phosphorylation in VEGF-treated cells. Other investigators have shown that leptin indirectly transactivates erbB2 [40] and stimulates epidermal growth factor (EGF) receptor phosphorylation in tumor cells [19]. Our novel observation that leptin's actions are mediated, at least in part, by ObRb-dependent VEGFR2 activation therefore lends support to the hypothesis that growth factor receptor activation is a key mechanism through which leptin regulates the angiogenic functions of ECs.

The results of this study clearly show that VEGFR2 plays a role in transducing pro-angiogenic signalling through the ObRb. Thus, leptin's ability to activate p38^MAPK^ and Akt, induce COX-2 expression, and stimulate neovascularisation of CAMs *in vivo* was suppressed by blocking the intrinsic tyrosine kinase activity of VEGFR2 with SU4516. In addition, the physiological relevance of VEGFR2 activation for leptin's pro-angiogenic actions is supported by our observations that leptin- as well as VEGF-stimulated EC proliferation, directional migration and differentiation *in vitro* were all abrogated by treatment with SU4516. Use of a neutralising antibody to VEGFR2 also substantially reduced the ability of both leptin and VEGF to stimulate tube formation on Matrigel, further confirming the importance of VEGFR2 and its activity as a mediator of leptin's effects on EC differentiation.

In ECs, up-regulation of COX-2 is generally associated with preferential release of the vasculoprotective mediator PGI_2_ [27]. We found that leptin enhanced release of both PGI_2_ and PGE_2_ from human ECs and, as expected, confirmed our previous studies demonstrating VEGF-stimulated prostanoid formation [17], [18]. It is unlikely, however, that rapid VEGFR2 activation by leptin results from immediate prostanoid release, since early prostanoid generation (<15 min) was not evident in leptin-stimulated ECs, and inhibition of COX-2 activity with NS398 had no effect on leptin-stimulated VEGFR2 phosphorylation (not shown). We have also shown that neither exogenous PGE_2_ [17] nor iloprost (a stable PGI_2_ analogue; not shown) stimulate VEGFR2 Tyr^1175^ phosphorylation in ECs, providing further support for the suggestion that leptin/VEGFR2 interactions are mainly upstream of prostanoid synthesis. The COX-2 products ultimately responsible for driving leptin's ability to enhance the angiogenic capabilities of ECs remain to be fully defined but both PGI_2_ and PGE_2_ are potential candidates since both are produced by leptin-stimulated cells and *in vivo* exposure of CAMs to iloprost or PGE_2_ accelerates their vascularisation (unpublished data).

Our studies also raise the interesting question of whether VEGFR2 is required for the full repertoire of leptin's actions on ECs. Although these have yet to be completely characterised it is evident that VEGF promotes von Willebrand factor secretion from human ECs [41] and enhances adhesion molecule expression [42], whereas leptin (1–100 ng/mL) does not (unpublished data). Thus, our current data suggest that ObRb-VEGFR2 crosstalk is restricted and may be utilised specifically to regulate leptin's pro-angiogenic actions.

Detrimental effects of leptin are well-documented and collectively support its involvement in obesity-driven vascular dysfunction. For example, leptin increases EC expression of pro-inflammatory/-thrombotic mediators [5], enhances platelet aggregation *in vitro* [28] and promotes inflammatory angiogenesis during tumour growth [31]. On the other hand, support for vasculoprotective actions of leptin is provided by observations that leptin phosphorylates and activates nitric oxide synthase (eNOS) [6], causes NO-dependent and -independent vasodilation [43], has direct cardioprotective and anti-atherogenic actions in mouse models [44] and improves vein graft performance in humans [45]. Our demonstration that leptin stimulates PGI_2_ formation may well suggest additional vasculoprotective roles and, since its angiogenic actions require COX-2 activity, raises the possibility that leptin could participate in reparative angiogenesis. Indeed, wound healing is impaired in leptin-deficient mice [46], leptin enhances the activity of circulating angiogenic cells [47] and a known vasculoprotective adipokine (adiponectin) improves hindlimb blood flow after ischaemia through mechanisms involving increased COX-2 activity [48].

In summary, we have shown that leptin binding to ObRb results in phosphorylation of VEGFR2, leading to activation of p38^MAPK^ and Akt, COX-2 induction and COX-2-dependent regulation of proliferation, motility and angiogenesis. Our studies identify VEGFR2 and COX-2 as key determinants of leptin-stimulated angiogenesis *in vitro* and *in vivo* and ObRb-dependent VEGFR2 activation reveals a new mode of leptin signalling in ECs which reinforces its importance as an angiogenic factor and establishes the functional significance of cross-talk with VEGF signalling. These findings are likely to have implications for leptin's regulation of endothelial cell function in both normal and obese individuals.

## Supporting Information

Figure S1 **Leptin enhances COX-2 mRNA expression in HUVEC.** Confluent quiescent HUVEC were challenged with vehicle alone, leptin (1–100 ng/mL) or VEGF (25 ng/mL) for 2, 4 and 6 hours. Total RNA was extracted and COX-2 and GAPDH mRNAs quantified by real-time reverse-transcription PCR (see Supplementary text S1). Each sample was analysed in triplicate. Results were normalised to GAPDH expression and are given as mean ± SEM (n = 4 individual experiments). *p<0.05 *versus* control.
(TIF)

Figure S2 **Leptin stimulates endothelial 6-keto-PGF_1α_ and PGE_2_ synthesis but not TxB_2_ formation.** Confluent cultures of HUVEC in 24-well trays were exposed to vehicle or leptin (1–100 ng/ml) for 8 hours (A and C) and 6 hours (B). Supernatants were collected and assayed for 6-keto-PGF_1α_ (A), PGE_2_ (B) and TxB_2_ (C) using commercially available assay kits (see Supplemetary text S1). The protein contents of whole cell lysates were quantified and eicosanoid synthesis calculated as pg/µg protein. Results are expressed as fold increases compared to time-matched controls (mean±SEM; triplicate observations in 3 separate experiments). Basal 6-keto-PGF_1α_ and PGE_2_ release were 47.3±40.6 and 2.3±1.2 pg/µg protein and in leptin-stimulated cells 1,082±312 and 6.3±2.5 pg/µg protein, respectively. *p<0.05 *versus* control.
(TIF)

Figure S3 **Effects of leptin on endothelial cell proliferation.** A and B. Sub-confluent HUVEC in 24-well trays were exposed to either vehicle (control), leptin (1–100 ng/mL) or VEGF (25 ng/mL) for 24 hours. Cells were then fixed, stained with propidium iodide (PI) and nuclei visualised using confocal microscopy. Each panel is representative of 12 images and each experiment was carried out in 4 individual cultures. C. Sub-confluent HUVEC in 96-well tissue culture trays were exposed to leptin (1–100 ng/mL) for 24 hours. Proliferation was assessed by measuring BrdU incorporation as described in Supplementary text S1. B. Subconfluent HUVEC in 96-well tissue culture trays were challenged with leptin (1–100 ng/mL) or VEGF (25 ng/mL), incubated overnight and calcein-AM was used to assess cell viability/proliferation (see Supplementary text S1). Data are expressed as mean± SEM (n = 4) with 4–6 observations per treatment (Panel B, C and D). *** *p*<0.001 *versus* control.
(TIF)

Figure S4 **Blockade of VEGFR2 activity with SU4516 attenuates leptin-driven endothelial cell proliferation and directional migration.** A: Subconfluent HUVEC were challenged with vehicle (control), leptin (1–100 ng/mL) or VEGF (25 ng/mL) in the absence or presence of SU4516 (5 µmol/L) for 24 hours. Nuclei were counted as described in Materials and Methods. B: Confluent cells were exposed to vehicle (control), leptin (1–100 ng/mL) or VEGF (25 ng/mL) in the absence or presence of SU4516 (5 µmol/L) and then scratch wounded. Migration was monitored by confocal microscopy. Data are expressed as mean ± SEM. * *p*<0.05 and *** *p*<0.001 *versus* control; # *p*<0.05 *versus* leptin treatment; § *p*<0.05 *versus* VEGF treatment.
(TIF)

Figure S5 **Leptin-stimulated endothelial cell differentiation on matrigel is inhibited by treatment with a VEGFR2 blocking antibody.** HUVEC (10,000 per well) were seeded onto 96-well plates coated with matrigel (50 µL/well) and treated with vehicle, leptin (100 ng/mL) or VEGF (25 ng/mL) in the presence or absence of a VEGFR2 blocking antibody or an IgG control antibody (100 ng/mL) for 8 hours. Cells were then fixed and imaged and the number of tubes/well quantified as described in Materials and Methods. Data are the mean of 2 individual experiments (duplicate observations per treatment).
(TIF)

Figure S6 **Leptin-stimulated VEGFR2 phosphorylation is not due to an association between VEGFR2 and ObRb.** VEGFR2 was immunoprecipitated from vehicle-, leptin (1 ng/mL)- or VEGF (25 ng/mL)-treated HUVEC (5 min) and immunoblots probed with antibodies against ObRb (A), VEGFR2 (B), or β-actin (C). UbP denotes unbound protein sample, demonstrating that ObRb was detected in ECs and that it was not co-immunoprecipitated with VEGFR2.
(TIF)

Text S1 (DOC)
