# Correction: Vascular Endothelial Growth Factor Receptor-2 Couples Cyclo-Oxygenase-2 with Pro-Angiogenic Actions of Leptin on Human Endothelial Cells

**DOI:** 10.1371/journal.pone.0223400

**Published:** 2019-09-30

**Authors:** Elena Garonna, Kathleen M. Botham, Graeme M. Birdsey, Anna M. Randi, Ruben R. Gonzalez-Perez, Caroline P. D. Wheeler-Jones

After publication of this article [1], concerns were raised about several western blot panels in Figs 1, 2, and 5.

Specifically, it was noted that there appear to be vertical discontinuities between lanes in some of the western blot panels, and questions were raised as to whether bands outlined in boxes originated from the same raw data image for each figure panel. The authors clarified that the data shown in each figure panel are taken from a single blot imaged at the same exposure; this is confirmed by the original image data, which are provided in S1–S4 Files. The black boxes around (or vertical lines between) lanes in the published figures indicate where original blot images were spliced for the purpose of presentation, i.e. to remove irrelevant lanes or rearrange data order. In some cases, these lines did not accurately reflect how figures were prepared, or were omitted in error in preparing the figures:

- Fig 1A: vertical lines ought to have been included in the COX-2 blot to indicate image splicing. Original blot data for this experiment are provided in S1 File.
- Fig 1B: a vertical line was errantly drawn on the total p38^mapk^ blot suggesting image splicing, but all lanes shown for this experiment were adjacent to one another on the original blot as is seen in the original blot image in S1 File.
- Fig 5D: There are vertical discontinuities between lanes 2/3 and 4/5 of the p-STAT3 blot, and between lanes 2/3 and 4/5 of the STAT3 blot. The vertical lines at the top of the panel demarcating ‘control’, ‘leptin’ and ‘VEGF’ incubations ought to have extended downwards through the p-VEGFR2, VEGFR2, p-STAT3 and STAT3 blots as the original blot images were spliced to rearrange data for the figure. Original data for the COX-1 experiment are no longer available; data supporting other panels are in S3 File. For the p-VEGFR2 experiment, it is unclear from the labelling on the original blot images whether the data shown in the figure for p-VEGFR2 and VEGFR2 (lanes highlighted in red in S3 File, “phospho and total VEGFR2 (Fig 5D) April 2019”) were obtained using the same samples.

The authors provide the following comments and clarifications:

- To address a brightness/contrast issue, a new scan of the Fig 2B COX-2 blot image is provided in S2 File.
- The word “COX-2” was omitted in error from the Fig 2B legend description of quantification data. The relevant sentence should read, “Analyses of COX-2 immunoblots from 3 separate experiments are shown.”
- In Fig 2C, p-Akt blot, lane 2 shows a 10 μM SB treatment rather than a 1 μM treatment as indicated in the legend. The original image underlying this figure shows results for both concentrations (S2 File, subfolder Fig 2C, “Fig 2C phospho-Akt scan of original blot”). The authors confirmed that the 1 μM data from lane 3 of the original blot were included in the quantification. Quantitative data are in S2 File, subfolder Fig 2C, “densitometry results”; this file includes summary-level data for this experiment rather than individual-level data for replicates from which the mean and standard error values were calculated.
- COX-1 was used as a protein loading control for several western blot experiments. The authors clarified that based on their previous [2, 3] and ongoing studies they have determined that COX-1 expression does not change following endothelial cell stimulation with the agonists examined (leptin, VEGF and thrombin). The uniformity of COX-1 expression across treatments shown in this manuscript provides additional confirmation of equal protein loading.
- Fig 5C does not include control blots showing unphosphorylated levels of p-GSK3β or p-Akt as controls for the signaling experiments. The authors commented that the total protein levels do not change following stimulation over the short time course used in these experiments, and that they had difficulty obtaining clear signal with GSK3β antibodies available at the time of the study. Hence they relied on COX-1 for a control given that it was found to have uniform expression across the incubation period examined in the Fig 5C phosphorylation experiments. Other figures of the article provide evidence that total Akt levels did not change following acute stimulation with the agonists used in the study.
- In Fig 1A-1C, the control and experimental data were obtained by running aliquots of the same experimental samples on parallel blots that were probed with the different antibodies. In Fig 1A, parallel blots were probed with COX-2 and COX-1 antibodies; in Fig 1B, parallel blots were probed with p-p38^mapk^ and p38^mapk^ antibodies; in Fig 1C, parallel blots were probed with COX-2 and COX-1 antibodies.
- S8–S11 Files provide additional information about available support for statements in the article referring to unpublished data or data not shown.

The original image data supporting blot panels in Fig 2A, COX-1 results in Fig 1C, total Akt results in Fig 2C, and phospho-p38 results in Fig 5C are no longer available. Quantitative data for Figs 3, 4, and 6 are in S5–S7 Files, and the underlying images for these results are available upon request from the corresponding author.

The authors apologize for the errors in the published article.

## Supporting information

S1 File Underlying data supporting Fig 1. (ZIP)

S2 File Underlying data supporting Fig 2. (ZIP)

S3 File Underlying data supporting Fig 5. (ZIP)

S4 File Original data supporting unpublished data statement 2. (PDF)

S5 File Original data supporting Fig 4 and unpublished data statement 6. (XLS)

S6 File Underlying data supporting Fig 3. (XLS)

S7 File Underlying data supporting Fig 6. (XLS)

S8 File Clarifications around statements in the article referring to unpublished data. (DOCX)

S9 File To examine the hypothesis that the degree of variation in HUVEC leptin sensitivity might relate to differences in leptin receptor (ObR) expression between cell isolates, unstimulated lysate samples from various HUVEC batches used in this and other studies were analyzed for ObR expression using western blotting. Densitometric analysis indicated that there might be a correlation, although weak, between ObR expression and the concentration of leptin that promoted the maximal response.
(XLS)

S10 File Underlying data supporting point 8: Iloprost does not stimulate VEGFR2 phosphorylation in ECs. (DOCX)

S11 File Underlying data supporting point 9: In vivo exposure of CAMs to prostaglandins increases vascularisation. (XLSX)
