# Supplementary figures and images for: Vascular Endothelial Growth Factor Receptor-2 Couples Cyclo-Oxygenase-2 with Pro-Angiogenic Actions of Leptin on Human Endothelial Cells
Source: PLoS One. 2011 Apr 18;6(4):e18823. doi: 10.1371/journal.pone.0018823 (PMC3078934; doi:10.1371/journal.pone.0018823)

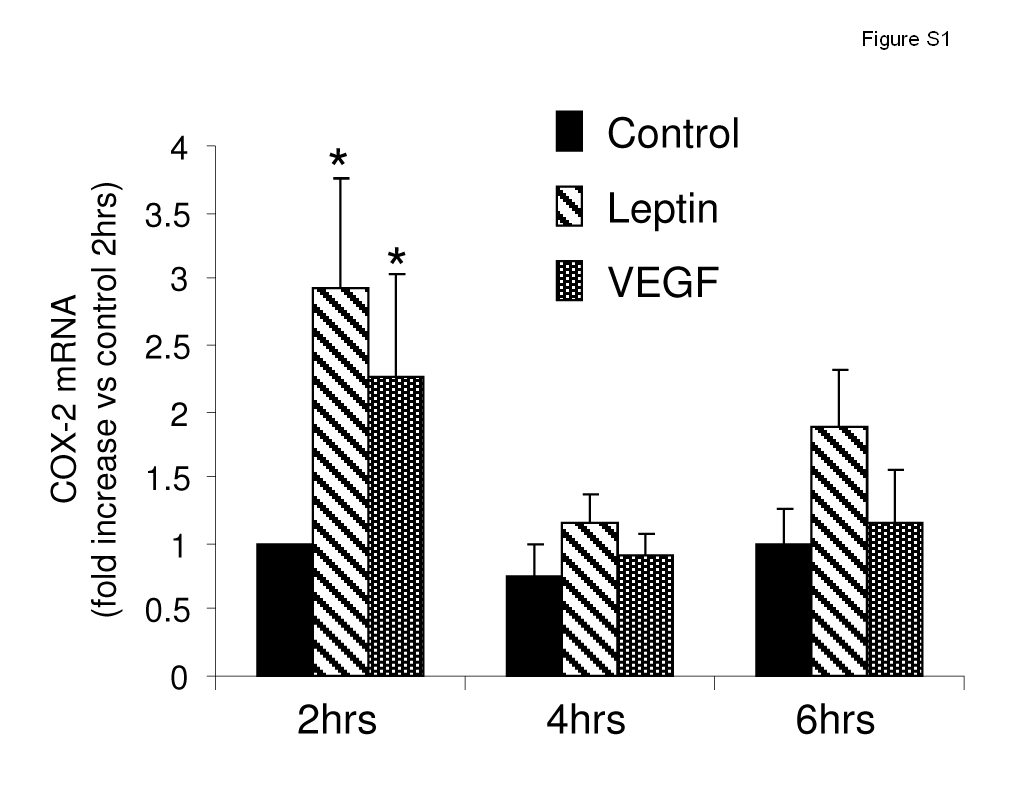

Supplement: Figure S1 — Leptin enhances COX-2 mRNA expression in HUVEC. Confluent quiescent HUVEC were challenged with vehicle alone, leptin (1–100 ng/mL) or VEGF (25 ng/mL) for 2, 4 and 6 hours. Total RNA was extracted and COX-2 and GAPDH mRNAs quantified by real-time reverse-transcription PCR (see Supplementary text S1). Each sample was analysed in triplicate. Results were normalised to GAPDH expression and are given as mean ± SEM (n = 4 individual experiments). *p<0.05 versus control. (TIF) [file pone.0018823.s001.tif]

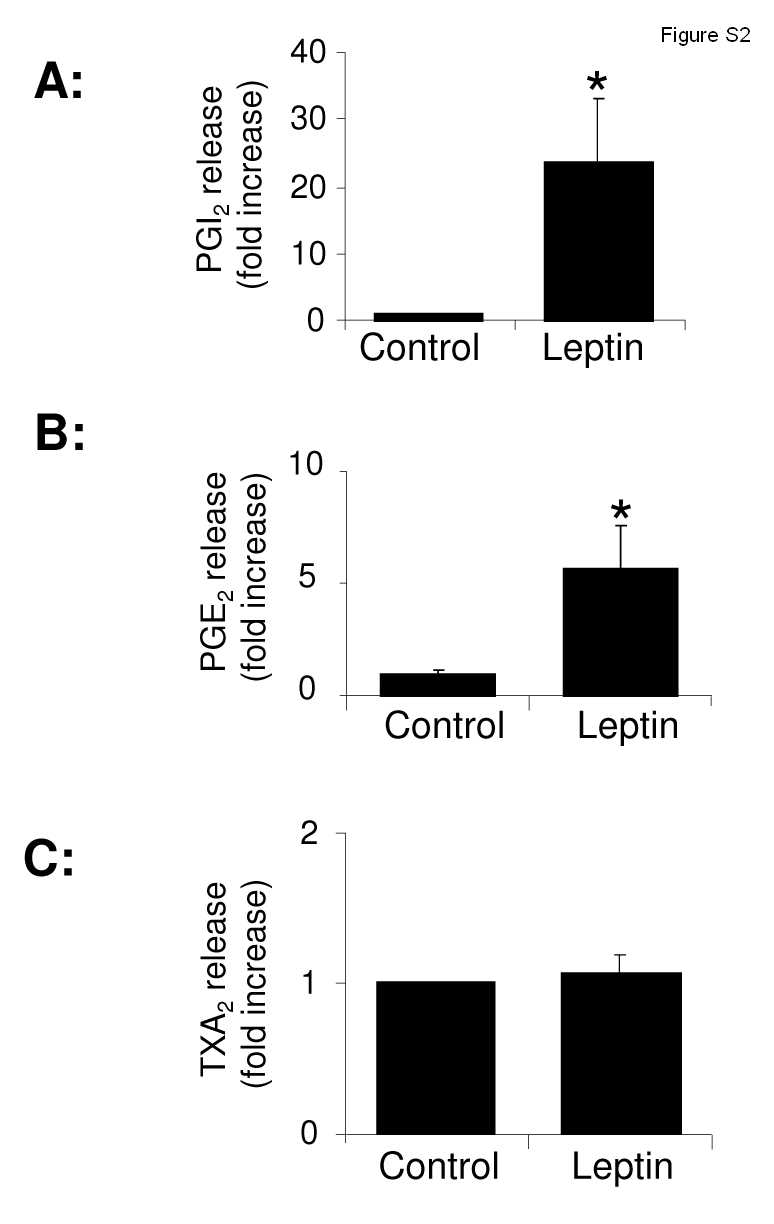

Supplement: Figure S2 — Leptin stimulates endothelial 6-keto-PGF1α and PGE2 synthesis but not TxB2 formation. Confluent cultures of HUVEC in 24-well trays were exposed to vehicle or leptin (1–100 ng/ml) for 8 hours (A and C) and 6 hours (B). Supernatants were collected and assayed for 6-keto-PGF1α (A), PGE2 (B) and TxB2 (C) using commercially available assay kits (see Supplemetary text S1). The protein contents of whole cell lysates were quantified and eicosanoid synthesis calculated as pg/µg protein. Results are expressed as fold increases compared to time-matched controls (mean±SEM; triplicate observations in 3 separate experiments). Basal 6-keto-PGF1α and PGE2 release were 47.3±40.6 and 2.3±1.2 pg/µg protein and in leptin-stimulated cells 1,082±312 and 6.3±2.5 pg/µg protein, respectively. *p<0.05 versus control. (TIF) [file pone.0018823.s002.tif]

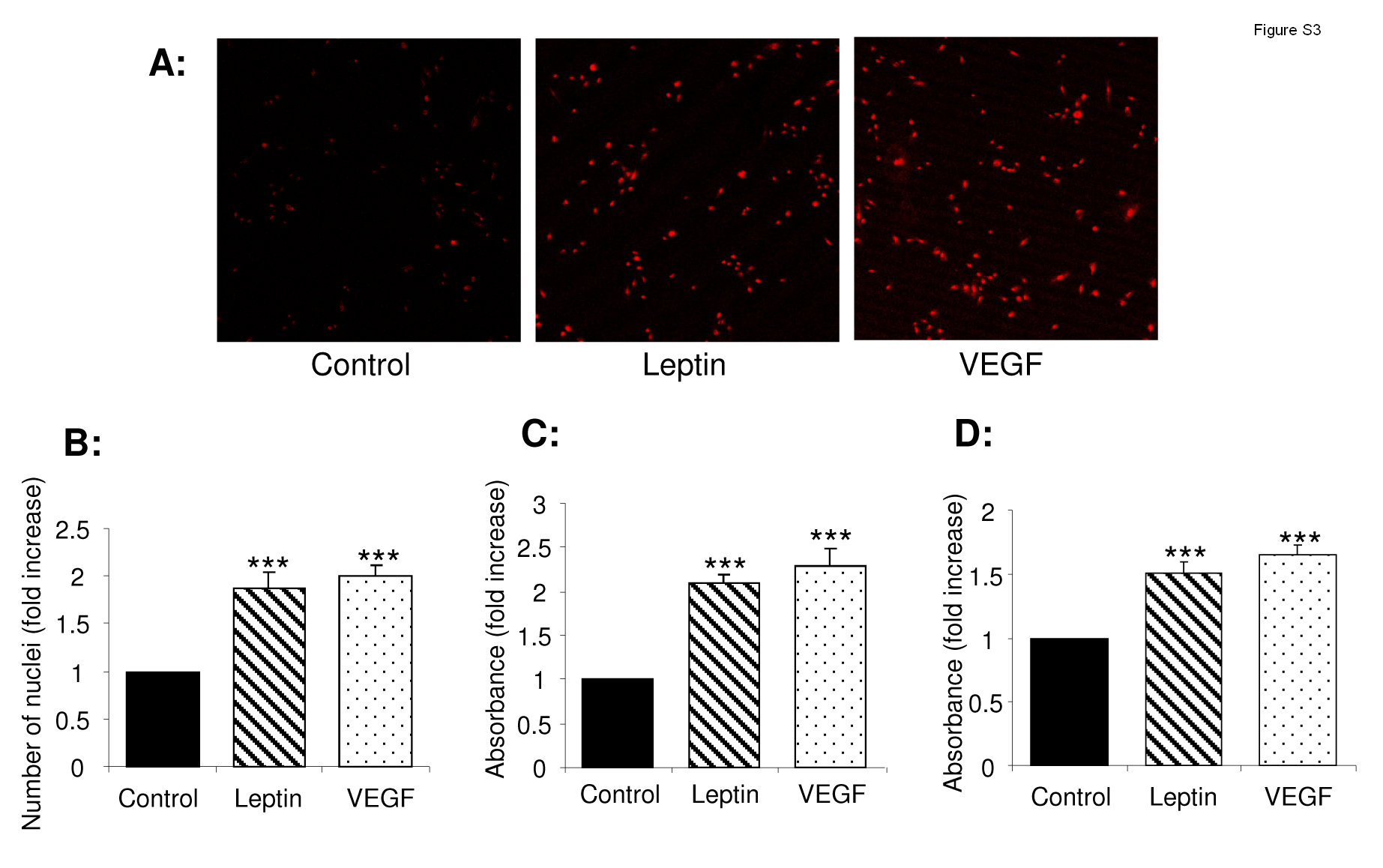

Supplement: Figure S3 — Effects of leptin on endothelial cell proliferation. A and B. Sub-confluent HUVEC in 24-well trays were exposed to either vehicle (control), leptin (1–100 ng/mL) or VEGF (25 ng/mL) for 24 hours. Cells were then fixed, stained with propidium iodide (PI) and nuclei visualised using confocal microscopy. Each panel is representative of 12 images and each experiment was carried out in 4 individual cultures. C. Sub-confluent HUVEC in 96-well tissue culture trays were exposed to leptin (1–100 ng/mL) for 24 hours. Proliferation was assessed by measuring BrdU incorporation as described in Supplementary text S1. B. Subconfluent HUVEC in 96-well tissue culture trays were challenged with leptin (1–100 ng/mL) or VEGF (25 ng/mL), incubated overnight and calcein-AM was used to assess cell viability/proliferation (see Supplementary text S1). Data are expressed as mean± SEM (n = 4) with 4–6 observations per treatment (Panel B, C and D). *** p<0.001 versus control. (TIF) [file pone.0018823.s003.tif]

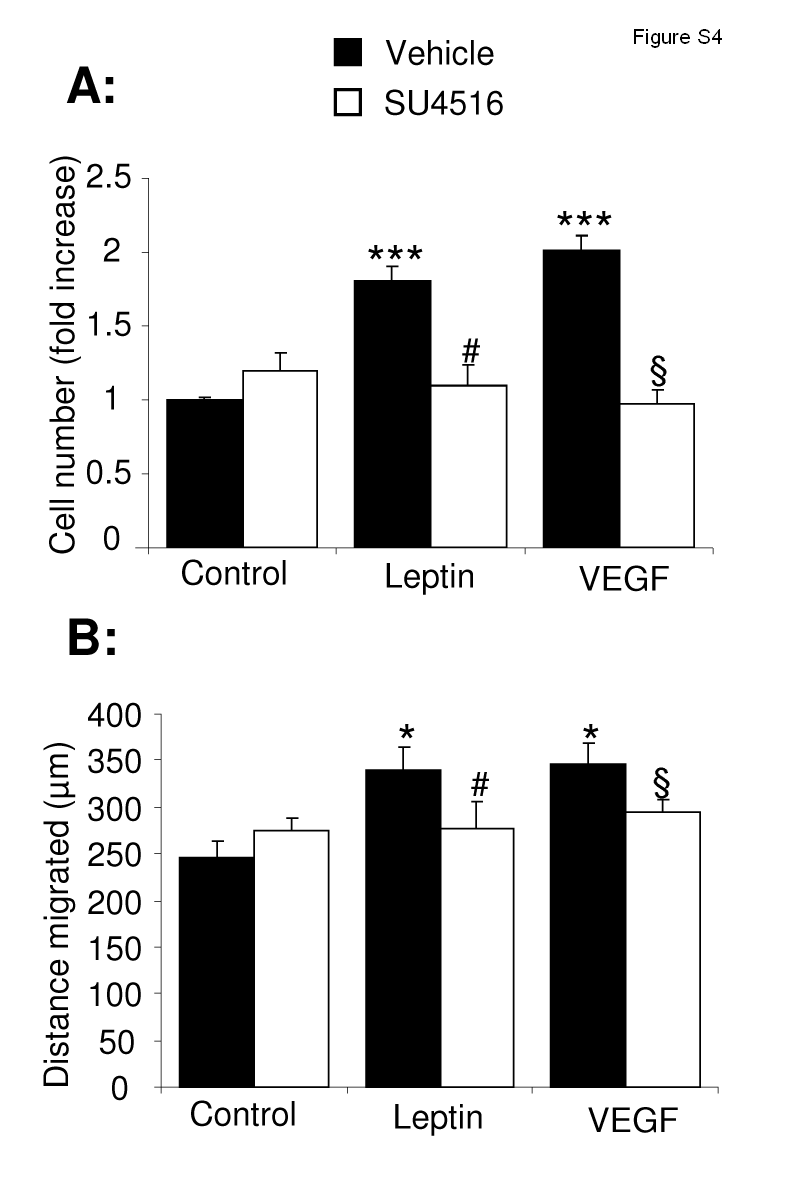

Supplement: Figure S4 — Blockade of VEGFR2 activity with SU4516 attenuates leptin-driven endothelial cell proliferation and directional migration. A: Subconfluent HUVEC were challenged with vehicle (control), leptin (1–100 ng/mL) or VEGF (25 ng/mL) in the absence or presence of SU4516 (5 µmol/L) for 24 hours. Nuclei were counted as described in Materials and Methods. B: Confluent cells were exposed to vehicle (control), leptin (1–100 ng/mL) or VEGF (25 ng/mL) in the absence or presence of SU4516 (5 µmol/L) and then scratch wounded. Migration was monitored by confocal microscopy. Data are expressed as mean ± SEM. * p<0.05 and *** p<0.001 versus control; # p<0.05 versus leptin treatment; § p<0.05 versus VEGF treatment. (TIF) [file pone.0018823.s004.tif]

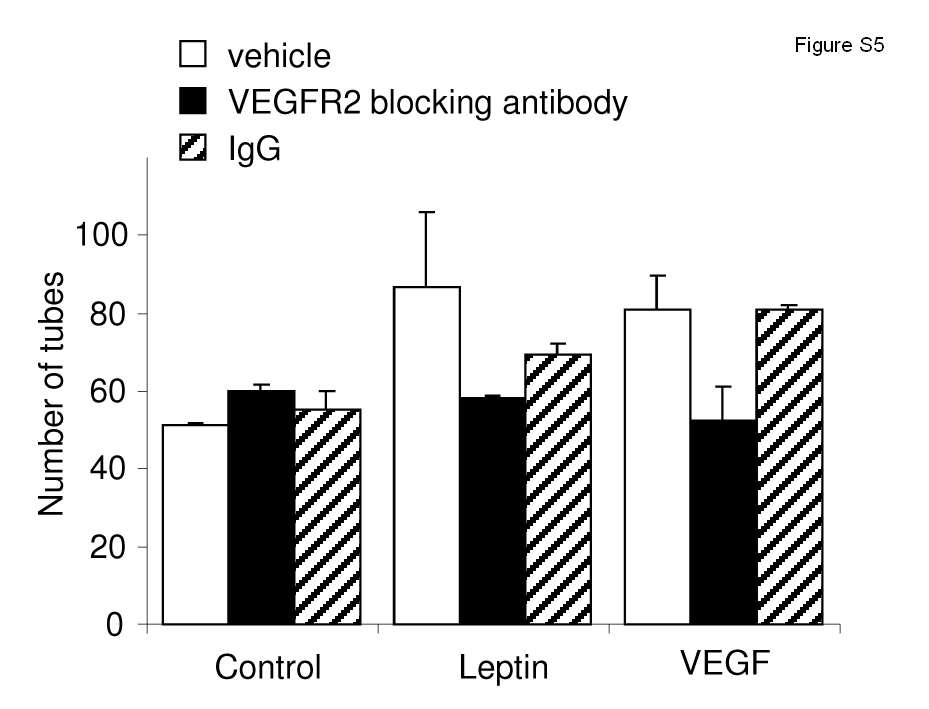

Supplement: Figure S5 — Leptin-stimulated endothelial cell differentiation on matrigel is inhibited by treatment with a VEGFR2 blocking antibody. HUVEC (10,000 per well) were seeded onto 96-well plates coated with matrigel (50 µL/well) and treated with vehicle, leptin (100 ng/mL) or VEGF (25 ng/mL) in the presence or absence of a VEGFR2 blocking antibody or an IgG control antibody (100 ng/mL) for 8 hours. Cells were then fixed and imaged and the number of tubes/well quantified as described in Materials and Methods. Data are the mean of 2 individual experiments (duplicate observations per treatment). (TIF) [file pone.0018823.s005.tif]

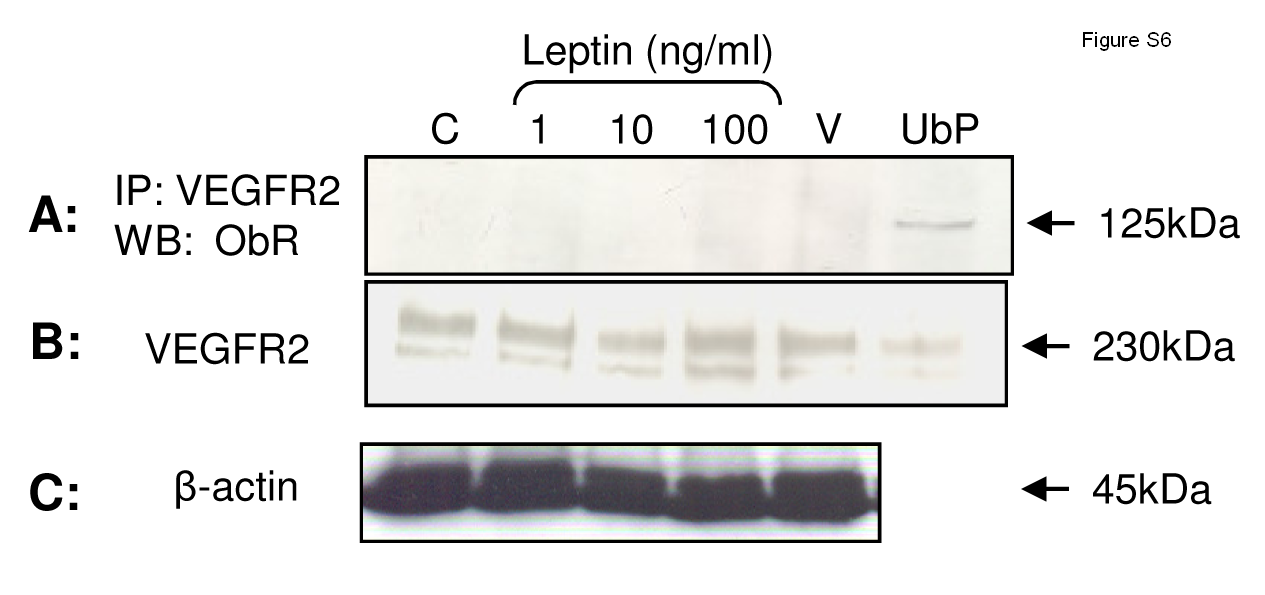

Supplement: Figure S6 — Leptin-stimulated VEGFR2 phosphorylation is not due to an association between VEGFR2 and ObRb. VEGFR2 was immunoprecipitated from vehicle-, leptin (1 ng/mL)- or VEGF (25 ng/mL)-treated HUVEC (5 min) and immunoblots probed with antibodies against ObRb (A), VEGFR2 (B), or β-actin (C). UbP denotes unbound protein sample, demonstrating that ObRb was detected in ECs and that it was not co-immunoprecipitated with VEGFR2. (TIF) [file pone.0018823.s006.tif]
