# Supplementary material for: Vascular Endothelial Growth Factor Receptor-2 Couples Cyclo-Oxygenase-2 with Pro-Angiogenic Actions of Leptin on Human Endothelial Cells
Source: PLoS One. 2011 Apr 18;6(4):e18823. doi: 10.1371/journal.pone.0018823 (PMC3078934; doi:10.1371/journal.pone.0018823)
Supplement: Text S1 — (DOC) [file pone.0018823.s007.doc]

**Supporting information**

*RNA isolation and quantitative real-time PCR (qPCR)*

Total RNA was isolated from quiescent HUVEC (serum- and ECGF-deprived) exposed to leptin or VEGF using an RNeasy Plus mini kit (Qiagen) according to the manufacturer’s instructions, and RNA yield and purity determined using a Nanodrop ND-1000 Fluorospectrometer. Procedures for reverse transcription of total RNA (1 µg) and subsequent qPCR analyses of COX-2 and GAPDH mRNA expression (DNA Engine Opticon 2; MJ Research Inc.) have been described previously .

*Measurement of eicosanoid synthesis*

ECs in 24-well trays were serum- and ECGF-deprived for 6-8 hours and exposed to leptin (1-100 ng/mL) for 8 hours. The PGI_2_, PGE_2_ and TxA_2_ contents of cell supernatants from control and leptin-stimulated cells were quantified using enzyme immunoassay kits (Assay Designs Inc) for 6-keto PGF_1α_, PGE_2_ and TxB_2_, respectively. Assays were carried out according to the manufacturers’ instructions.

*Measurement of cell viability/proliferation using calcein AM*

HUVEC viability/proliferation was assessed using calcein acetoxymethyl (calcein AM). ECs were seeded in 96-well plates (10,000/well), cultured in full medium M199 for 8-14 hours and then treated as described in the figure legends. Following overnight incubation, the medium was aspirated and replaced with calcein AM (0.4 μM; 200 μL/well) prepared in serum-free medium. Following incubation for 30 minutes at 37°C cells were washed in serum-free medium and then lysed in 100 μL of lysis buffer (150 mM NaCl, 10 mM Tris HCl, 5 mM EDTA, 1% NP-40, 1 mM PMSF, 50 μg/mL leupeptin). Fluorescence was monitored at λ_ex485_ λ_em530 (535)_ on a Wallac Victor^2^ 1420 multilabel counter.

*Measurement of 5-bromo-2’-deoxyuridine (BrdU) incorporation*

The proliferative capacity of HUVEC (passage 1) was determined using a BrdU ELISA cell proliferation assay according to the manufacturer’s instructions. Cells were seeded at a density of 10,000/well in 96-well plates and cultured for 6 hrs. Following serum and ECGF deprivation (12-16 hours), cells were treated with the appropriate stimulant for 24 hours and BrdU (final concentration 10 µmol/L) was added to the cultures for the last 5 hours of incubation. Cells were then fixed and DNA denatured, and a peroxidase-labelled anti-BrdU antibody (anti-BrdU-POD antibody, Fab fragments; 100 μL/well) was added to the wells for 90 minutes. The absorbance was measured directly using a spectrophotometric microplate reader (Wallac Victor^2^ 1420 multilabel counter) at a test wavelength of 450 nm and a reference wavelength of 490 nm and was directly proportional to the amount of BrdU incorporation.

**References**

1. Syeda F, Grosjean J, Houliston RA, Keogh RJ, Carter TD, et al. (2006) Cyclooxygenase-2 induction and prostacyclin release by protease-activated receptors in endothelial cells require cooperation between mitogen-activated protein kinase and NF-kappaB pathways. J Biol Chem 281: 11792-11804.

2. Lynch CN, Wang YC, Lund JK, Chen Y-W, Leal JA, et al. (1999) TWEAK Induces Angiogenesis and Proliferation of Endothelial Cells. J Biol Chem 274: 8455-8459.
