# Supplementary material for: Correction: Vascular Endothelial Growth Factor Receptor-2 Couples Cyclo-Oxygenase-2 with Pro-Angiogenic Actions of Leptin on Human Endothelial Cells
Source: PLoS One. 2019 Sep 30;14(9):e0223400. doi: 10.1371/journal.pone.0223400 (PMC6768471; doi:10.1371/journal.pone.0223400)
Supplement: S1 File — (ZIP) [file pone.0223400.s001.zip › Figure 1/Fig.1A/Fig.1A COX-1 blot scan.docx]

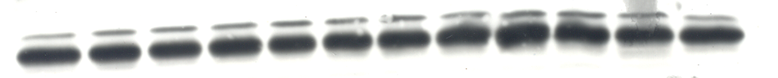


1 2 3

Original scan image for representative COX-1 blot in Figure 1A lower panel.

Lanes 1 (control), 2 (leptin) and 3 (VEGF) are shown in Figure 1A (left to right).
