# Supplementary material for: Correction: Vascular Endothelial Growth Factor Receptor-2 Couples Cyclo-Oxygenase-2 with Pro-Angiogenic Actions of Leptin on Human Endothelial Cells
Source: PLoS One. 2019 Sep 30;14(9):e0223400. doi: 10.1371/journal.pone.0223400 (PMC6768471; doi:10.1371/journal.pone.0223400)
Supplement: S1 File — (ZIP) [file pone.0223400.s001.zip › Figure 1/Fig.1B/Fig.1B phospho-p38 original scan of blot (uncropped).docx]

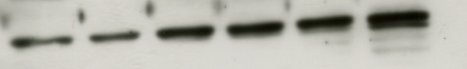


1 5 6

Original uncropped scan of representative phospho-p38^mapk^ blot in Fig.1B (upper panel)

Lanes 1 (control), 5 (leptin) and 6 (VEGF) are shown in Fig.1B.
