# Supplementary material for: Correction: Vascular Endothelial Growth Factor Receptor-2 Couples Cyclo-Oxygenase-2 with Pro-Angiogenic Actions of Leptin on Human Endothelial Cells
Source: PLoS One. 2019 Sep 30;14(9):e0223400. doi: 10.1371/journal.pone.0223400 (PMC6768471; doi:10.1371/journal.pone.0223400)
Supplement: S1 File — (ZIP) [file pone.0223400.s001.zip › Figure 1/Fig.1B/Fig.1B total p38 scan of original blot.docx]

1 2 3 4 5 6 7 8 9 10 11 12 13

Scan of original representative total p38 blot (Fig.1B lower panel)

Lanes 6 (control), 7 (leptin) and 8 (VEGF) are given in Fig.1B.
