# Supplementary material for: Correction: Vascular Endothelial Growth Factor Receptor-2 Couples Cyclo-Oxygenase-2 with Pro-Angiogenic Actions of Leptin on Human Endothelial Cells
Source: PLoS One. 2019 Sep 30;14(9):e0223400. doi: 10.1371/journal.pone.0223400 (PMC6768471; doi:10.1371/journal.pone.0223400)
Supplement: S1 File — (ZIP) [file pone.0223400.s001.zip › Figure 1/Fig.1C/Fig.1C COX-2 scan of original blot.docx]

1 3 5 7 9 11

Scan of original representative COX-2 blot (Fig.1C upper panel)

Lanes 1, 3, 5, 7, 9 and 11 are shown in Fig.1C

1: Control

3: SB alone (1uM)

5: Leptin

7: Leptin + SB

9: VEGF

11:VEGF + SB
