# Supplementary figures and images for: Correction: Vascular Endothelial Growth Factor Receptor-2 Couples Cyclo-Oxygenase-2 with Pro-Angiogenic Actions of Leptin on Human Endothelial Cells
Source: PLoS One. 2019 Sep 30;14(9):e0223400. doi: 10.1371/journal.pone.0223400 (PMC6768471; doi:10.1371/journal.pone.0223400)

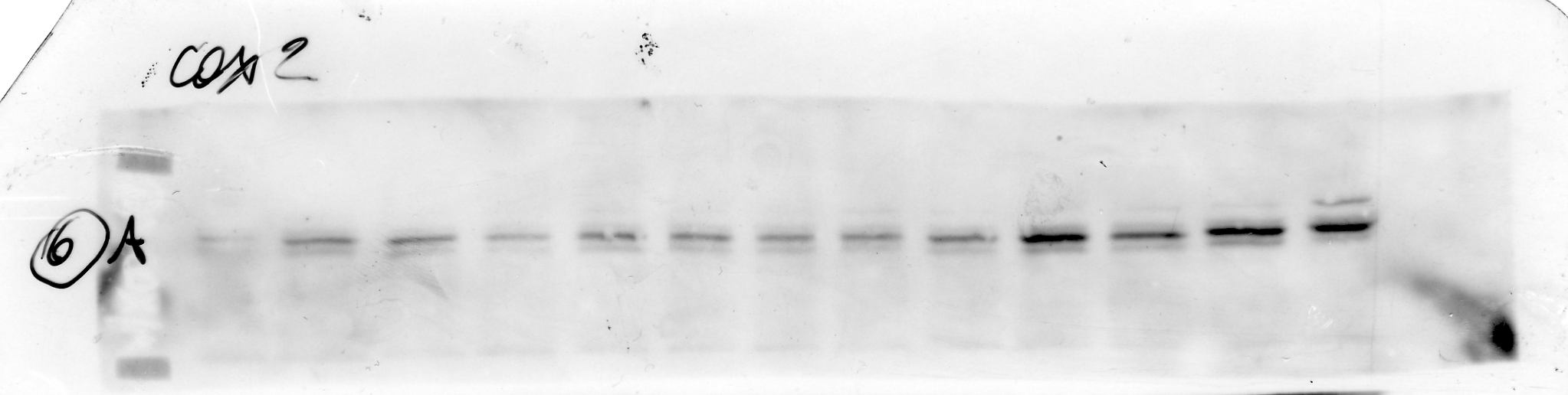

Supplement: S1 File — (ZIP) [file pone.0223400.s001.zip › Figure 1/Fig.1A/MSc exp 6.tif]

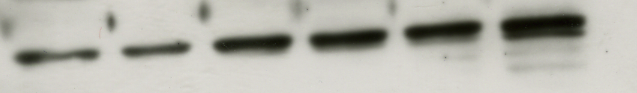

Supplement: S1 File — (ZIP) [file pone.0223400.s001.zip › Figure 1/Fig.1B/exp 18 leptin p38.tif]

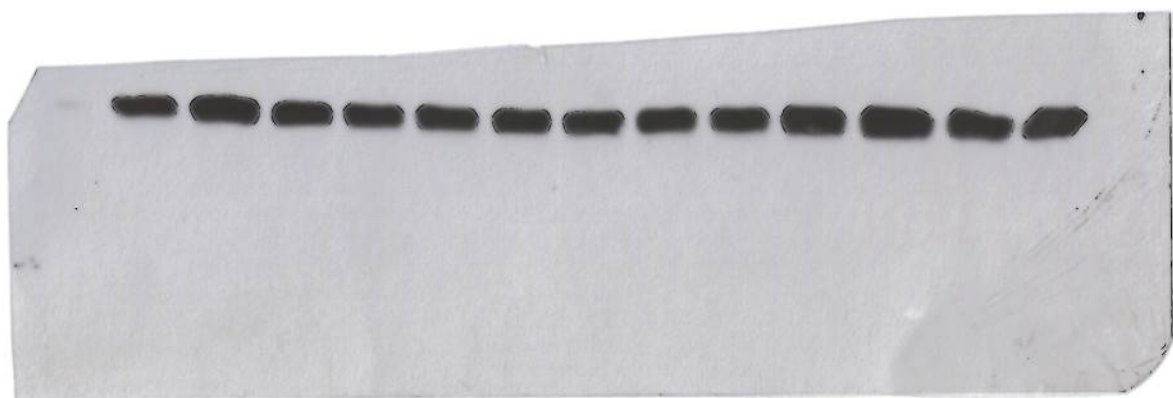

Supplement: S1 File — (ZIP) [file pone.0223400.s001.zip › Figure 1/Fig.1B/Fig.1B (lower panel) original scan of blot.pdf]

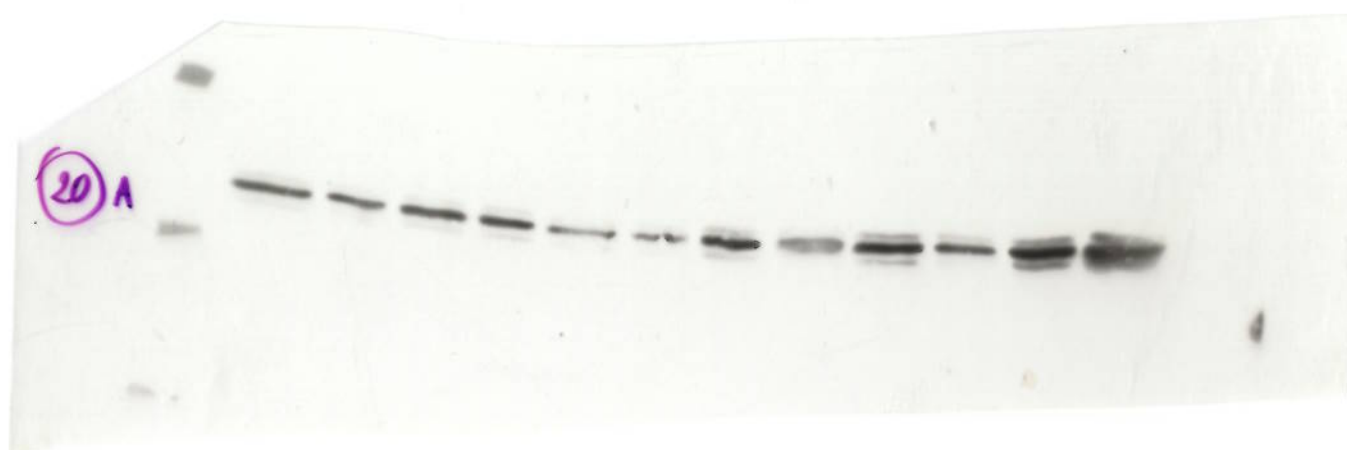

Supplement: S2 File — (ZIP) [file pone.0223400.s002.zip › Figure 2/Fig.2B/Fig 2B scan of original blot.pdf]

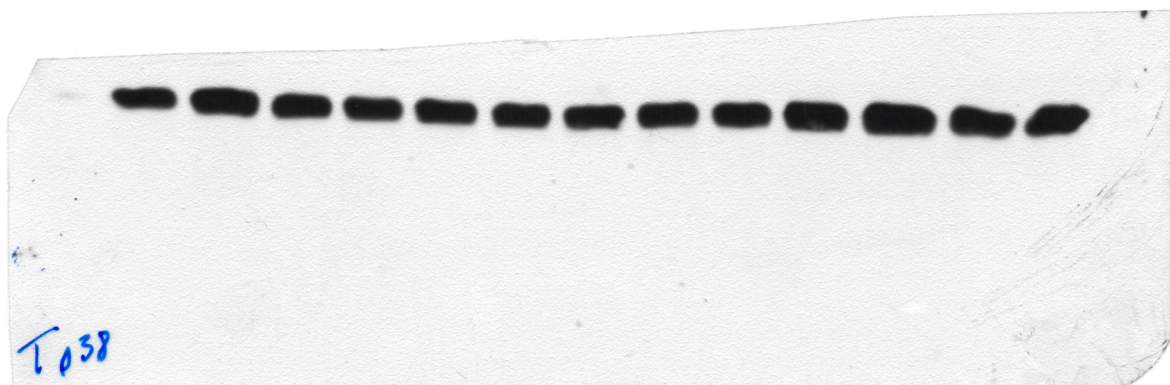

Supplement: S4 File — (PDF) [file pone.0223400.s004.pdf]
