# Supplementary material for: Correction: Vascular Endothelial Growth Factor Receptor-2 Couples Cyclo-Oxygenase-2 with Pro-Angiogenic Actions of Leptin on Human Endothelial Cells
Source: PLoS One. 2019 Sep 30;14(9):e0223400. doi: 10.1371/journal.pone.0223400 (PMC6768471; doi:10.1371/journal.pone.0223400)
Supplement: S2 File — (ZIP) [file pone.0223400.s002.zip › Figure 2/Fig.2B/Scan of COX-1 blot (Fig 2B).docx]

1 2 3 4 5 6 7 8 9 10 11 12 13


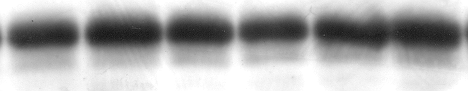


Scan of original total COX-1 blot (Fig.2B) with a slightly longer exposure time. The smaller image below is the cropped image shown in Fig.2B with lanes as below.

6 (control), 7 (LY), 8 (leptin) and 9 (leptin plus LY).
