# Supplementary material for: Correction: Vascular Endothelial Growth Factor Receptor-2 Couples Cyclo-Oxygenase-2 with Pro-Angiogenic Actions of Leptin on Human Endothelial Cells
Source: PLoS One. 2019 Sep 30;14(9):e0223400. doi: 10.1371/journal.pone.0223400 (PMC6768471; doi:10.1371/journal.pone.0223400)
Supplement: S2 File — (ZIP) [file pone.0223400.s002.zip › Figure 2/Fig.2B/Scan of COX-2 blot (Fig 2B).docx]

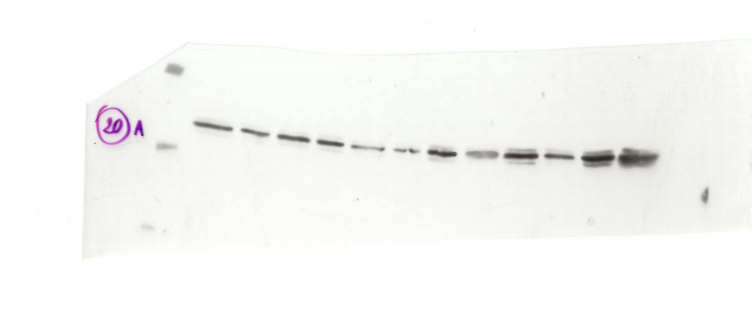


1 2 3 4 5 6 7 8 9 10 11 12

Re-scan of original blot shown in Fig 2B

Lanes 7 to 10 (left to right) are included in Fig 2B with treatments (6h) as indicated in the manuscript:

7: control

8: LY alone

9: leptin

10:leptin plus LY

(11: Thrombin

12: thrombin plus LY

Lanes 1-6 are the same experimental treatments for 30 min ~ COX-2 protein is not induced at 30 min)
