# Supplementary material for: Correction: Vascular Endothelial Growth Factor Receptor-2 Couples Cyclo-Oxygenase-2 with Pro-Angiogenic Actions of Leptin on Human Endothelial Cells
Source: PLoS One. 2019 Sep 30;14(9):e0223400. doi: 10.1371/journal.pone.0223400 (PMC6768471; doi:10.1371/journal.pone.0223400)
Supplement: S2 File — (ZIP) [file pone.0223400.s002.zip › Figure 2/Fig.2C/Fig.2C phospho-Akt scan of original blot.docx]

1 2 3 4 5 6 7 8 9 10 11 12

Scan of original representative full phospho-Akt blot (Fig.2C)

Lanes 1, 3 , 5 and 7 are shown in Fig.2C.

1: control

2: SB 0.1µM

3: SB 1µM

4: SB 10 µM

5: leptin

6: leptin plus SB 0.1µM

7: leptin plus SB 1µM

8: leptin plus SB 10µM

(Lanes 9-12 are the same treatments with thrombin)
