# Supplementary material for: Correction: Vascular Endothelial Growth Factor Receptor-2 Couples Cyclo-Oxygenase-2 with Pro-Angiogenic Actions of Leptin on Human Endothelial Cells
Source: PLoS One. 2019 Sep 30;14(9):e0223400. doi: 10.1371/journal.pone.0223400 (PMC6768471; doi:10.1371/journal.pone.0223400)
Supplement: S3 File — (ZIP) [file pone.0223400.s003.zip › Figure 5/Fig.5A/Fig.5A phospho-Tyr scan of original blot.docx]

1 2 3 4 5 6

Scan of original phospho-Tyr blot (Fig.5A upper panel)

Lanes 3, 4 and 5 are shown in Fig.5A in the manuscript.

1: control

2: leptin

3: control

4: leptin

5: VEGF

6: VEGF
