# Supplementary material for: Correction: Vascular Endothelial Growth Factor Receptor-2 Couples Cyclo-Oxygenase-2 with Pro-Angiogenic Actions of Leptin on Human Endothelial Cells
Source: PLoS One. 2019 Sep 30;14(9):e0223400. doi: 10.1371/journal.pone.0223400 (PMC6768471; doi:10.1371/journal.pone.0223400)
Supplement: S3 File — (ZIP) [file pone.0223400.s003.zip › Figure 5/Fig.5C/Phospho-Akt (Fig 5C).docx]

1 2 3 4 5 6 7 8 9 10 11 12 13


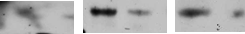


Control leptin VEGF

- + - + - + SU4516

Original pAkt blot plus original sections of scanned blot in Fig.5C showing the pairs of experimental treatments separated from each other (these are separated by a vertical line in the manuscript).
