# Supplementary material for: Correction: Vascular Endothelial Growth Factor Receptor-2 Couples Cyclo-Oxygenase-2 with Pro-Angiogenic Actions of Leptin on Human Endothelial Cells
Source: PLoS One. 2019 Sep 30;14(9):e0223400. doi: 10.1371/journal.pone.0223400 (PMC6768471; doi:10.1371/journal.pone.0223400)
Supplement: S3 File — (ZIP) [file pone.0223400.s003.zip › Figure 5/Fig.5D/COX (Fig 5D).docx]

1 2 3 4 5 6 7 8 9 10 11 12 13 14


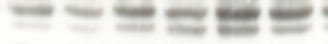


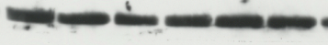


Scan of full original COX-2 blot (Fig.5D). Lanes 5-10 are shown in Fig.5D in the manuscript.

The 2 panels below show the images presented in the manuscript (upper COX-2; lower corresponding COX-1)

5: control

6: peptide alone

7: Leptin

8: Leptin plus peptide

9: VEGF

10: VEGF plus peptide
