# Supplementary material for: Correction: Vascular Endothelial Growth Factor Receptor-2 Couples Cyclo-Oxygenase-2 with Pro-Angiogenic Actions of Leptin on Human Endothelial Cells
Source: PLoS One. 2019 Sep 30;14(9):e0223400. doi: 10.1371/journal.pone.0223400 (PMC6768471; doi:10.1371/journal.pone.0223400)
Supplement: S3 File — (ZIP) [file pone.0223400.s003.zip › Figure 5/Fig.5D/phospho and total VEGFR2 (Fig 5D).docx]

1 2 3 4 5 6 7 8 9 10 11 12 13 14

Original p-VEGFR2 and total VEGFR2 blots (Fig.5D) indicating pairs of experimental treatments shown in the manuscript.

1: control

2: peptide alone

5: Leptin

6: Leptin plus peptide

7: VEGF

8: VEGF plus peptide
