# Supplementary material for: Correction: Vascular Endothelial Growth Factor Receptor-2 Couples Cyclo-Oxygenase-2 with Pro-Angiogenic Actions of Leptin on Human Endothelial Cells
Source: PLoS One. 2019 Sep 30;14(9):e0223400. doi: 10.1371/journal.pone.0223400 (PMC6768471; doi:10.1371/journal.pone.0223400)
Supplement: S3 File — (ZIP) [file pone.0223400.s003.zip › Figure 5/Fig.5D/phospho-Stat3 (Fig 5D).docx]

1 2 3 4 5 6 7 8 9 10 11 12 13 14

Scan of original phospho-STAT-3 blot (Fig.5D)

Lanes 4&5, 8 &9 and 13&14 are shown in Fig.5D in the manuscript (see below the original images used in the manuscript separated to show the pairs of experimental treatments)


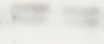

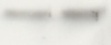

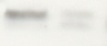


4: leptin

5: leptin plus peptide

8: control

9: peptide alone

13: VEGF

14: VEGF plus peptide
