# Supplementary material for: Correction: Vascular Endothelial Growth Factor Receptor-2 Couples Cyclo-Oxygenase-2 with Pro-Angiogenic Actions of Leptin on Human Endothelial Cells
Source: PLoS One. 2019 Sep 30;14(9):e0223400. doi: 10.1371/journal.pone.0223400 (PMC6768471; doi:10.1371/journal.pone.0223400)
Supplement: S3 File — (ZIP) [file pone.0223400.s003.zip › Figure 5/Fig.5D/Total Stat3 (Fig 5D).docx]

1 2 3 4 5 6 7 8 9 10 11 12 13 14

Scan of original total STAT-3 blot (Fig.5D)

Lanes 7-12 are shown in Fig.5D in the manuscript.

4: control

5: peptide alone

8: Leptin

9: Leptin plus peptide

13: VEGF

14: VEGF plus peptide
