# Supplementary material for: Correction: Vascular Endothelial Growth Factor Receptor-2 Couples Cyclo-Oxygenase-2 with Pro-Angiogenic Actions of Leptin on Human Endothelial Cells
Source: PLoS One. 2019 Sep 30;14(9):e0223400. doi: 10.1371/journal.pone.0223400 (PMC6768471; doi:10.1371/journal.pone.0223400)
Supplement: S8 File — (DOCX) [file pone.0223400.s008.docx]

“Data not shown” and “unpublished data” are referenced several times in the article. In two cases (points 6, 10 below), the supporting data underlying the statements are not available. The relevant sentences in the article and availability or unavailability of supporting data are noted here:

- 1. “The leptin concentration evoking a maximal effect on the signalling and functional responses investigated differed among HUVEC isolates (data not shown)”
     - Evidence that there can be variation in leptin sensitivity in cells isolated from individual cords, and relationship to the level of leptin receptor expression as assessed by western blotting is shown in File S8.
  2. “Leptin enhanced p38^MAPK^ phosphorylation ([Figure 1B](https://journals.plos.org/plosone/article?id=10.1371/journal.pone.0018823#pone-0018823-g001)) in a time-dependent manner (data not shown) with maximal activation evident after 10 minutes. “
     - File S4 shows an example of a preliminary experiment to determine the optimum stimulation time-point.
  3. “p38^MAPK^ inhibitor SB202190 concentration-dependently decreased COX-2 protein expression in leptin- and thrombin-stimulated cells ([Figure 1C](https://journals.plos.org/plosone/article?id=10.1371/journal.pone.0018823#pone-0018823-g001); concentration data not shown). “
     - The data shown in Figure 1C show the effects of the maximally effective concentration of SB202190. An example of a COX-2 blot showing the effects of different concentrations of SB202190 on leptin- and thrombin-stimulated cells is in File S1 (Fig.1C COX-2 scan of original blot with labels), but the control data (COX-1 blot) for this experiment are no longer available.
  4. “In keeping with our recent findings [[26]](https://journals.plos.org/plosone/article?id=10.1371/journal.pone.0018823#pone.0018823-Hamdulay1), [[27]](https://journals.plos.org/plosone/article?id=10.1371/journal.pone.0018823#pone.0018823-Syeda1), HUVEC exhibited some basal COX-2 expression which was also reduced by SB202190 treatment (data not shown).“
     - The statement ‘data not shown’ should not have been included here and was added in error. Figure 1C shows that basal COX-2 expression is significantly reduced by SB202190.
  5. “Akt phosphorylation was enhanced in cells exposed to leptin ([Figure 2A](https://journals.plos.org/plosone/article?id=10.1371/journal.pone.0018823#pone-0018823-g002)) and LY294002, a PI3K inhibitor which blocks downstream Akt phosphorylation (data not shown).”
     - The statement ‘data not shown’ was included here in error.
  6. “SU4516 (5 µmol/L) inhibited leptin- and VEGF-induced proliferation but did not modify basal ([Figure S4](https://journals.plos.org/plosone/article?id=10.1371/journal.pone.0018823#pone.0018823.s004)) or hepatocyte growth factor (HGF)-induced proliferation (data not shown).”
     - Data supporting this statement are not available, but the authors have provided data for related experiments demonstrating that SU5416 did not affect HGF-induced tube formation (tabs Exp 73 and Exp 83 tabs in File S5) or GSK3β phosphorylation (File [Phospho-GSK3b.doc] in Fig 5C subfolder of File S3).
  7. “Early prostanoid generation (<15 min) was not evident in leptin-stimulated ECs, and inhibition of COX-2 activity with NS398 had no effect on leptin-stimulated VEGFR2 phosphorylation (not shown).”
     - Underlying data supporting this statement are not available.
  8. “Neither exogenous PGE_2_ [[17]](https://journals.plos.org/plosone/article?id=10.1371/journal.pone.0018823#pone.0018823-Clarkin1) nor iloprost (a stable PGI_2_ analogue; not shown) stimulate VEGFR2 Tyr^1175^ phosphorylation in ECs.”
     - Data supporting this statement are in File S9.
  9. “…both PGI2 and PGE2 are potential candidates since both are produced by leptin-stimulated cells and in vivo exposure of CAMs to iloprost or PGE2 accelerates their vascularisation (unpublished data).”

Data supporting this statement are in File S10.

- 1. “Although these have yet to be completely characterised it is evident that VEGF promotes von Willebrand factor secretion from human ECs [41] and enhances adhesion molecule expression [42], whereas leptin (1–100 ng/mL) does not (unpublished data).”
     - Data supporting this statement are not available.
