# Supplementary material for: Correction: Vascular Endothelial Growth Factor Receptor-2 Couples Cyclo-Oxygenase-2 with Pro-Angiogenic Actions of Leptin on Human Endothelial Cells
Source: PLoS One. 2019 Sep 30;14(9):e0223400. doi: 10.1371/journal.pone.0223400 (PMC6768471; doi:10.1371/journal.pone.0223400)
Supplement: S10 File — (DOCX) [file pone.0223400.s010.docx]

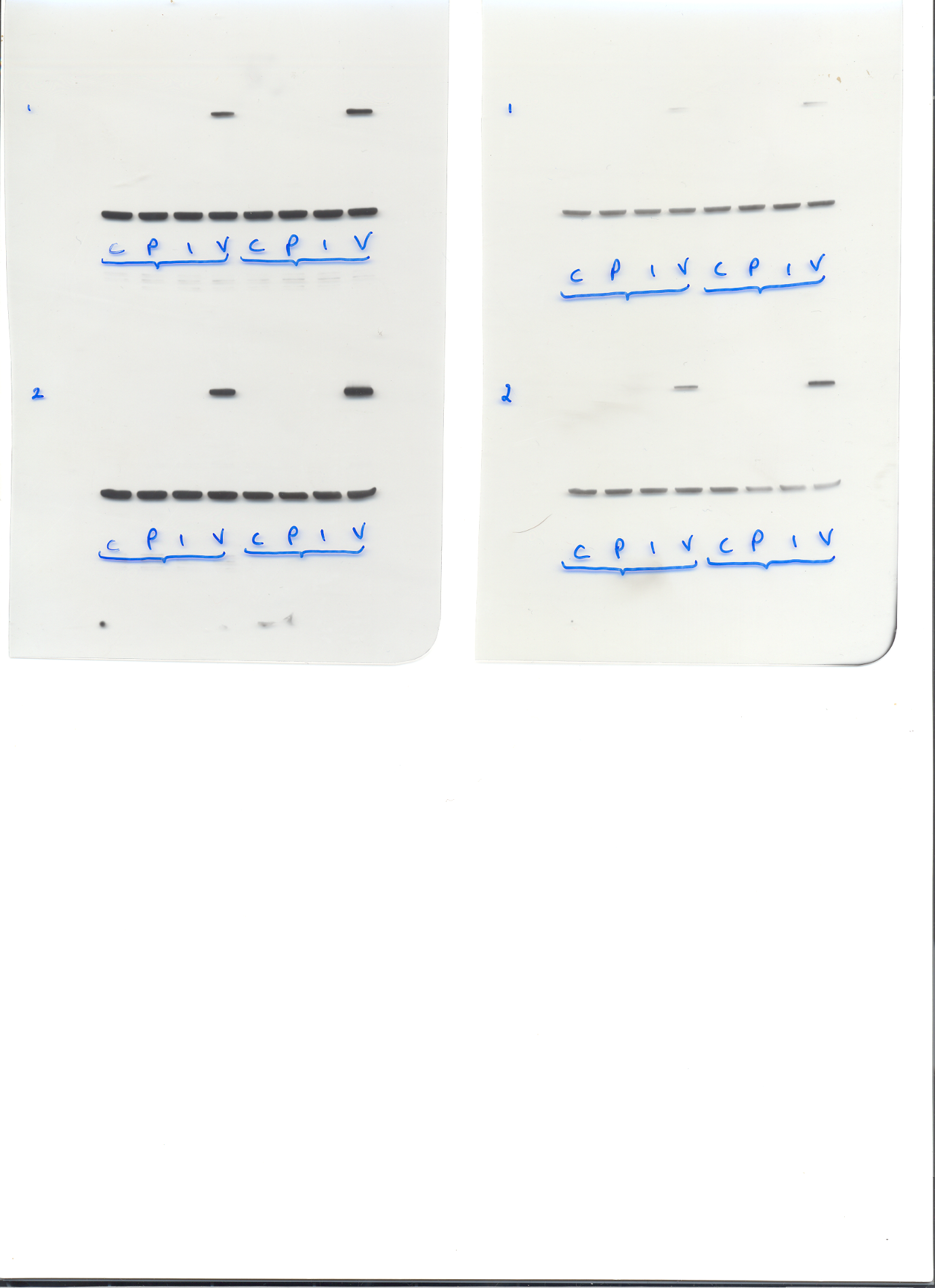


Data supporting point 8 in your Word document. These two individual experiments (1 and 2; carried out in 2010) show that neither iloprost (I) nor PGE_2_ (P) stimulate phosphorylation of VEGFR2 on Tyr^1175^, whereas VEGF (V) does.

Beta-actin

Beta-actin

pVEGR2

pVEGR2

The blots were probed with anti-pVEGFR2 and with anti-beta actin to confirm equal protein loading.
